# Sulfide Homeostasis and Nitroxyl Intersect via Formation of Reactive Sulfur Species in *Staphylococcus aureus*

**DOI:** 10.1128/mSphere.00082-17

**Published:** 2017-06-21

**Authors:** Hui Peng, Jiangchuan Shen, Katherine A. Edmonds, Justin L. Luebke, Anne K. Hickey, Lauren D. Palmer, Feng-Ming James Chang, Kevin A. Bruce, Thomas E. Kehl-Fie, Eric P. Skaar, David P. Giedroc

**Affiliations:** a Department of Chemistry, Indiana University, Bloomington, Indiana, USA; b Graduate Program in Biochemistry, Indiana University, Bloomington, Indiana, USA; c Department of Molecular and Cellular Biochemistry, Indiana University, Bloomington, Indiana, USA; d Department of Biology, Indiana University, Bloomington, Indiana, USA; e Department of Pathology, Microbiology, and Immunology, Vanderbilt University Medical Center, Nashville, Tennessee, USA; f Department of Microbiology, University of Illinois Urbana–Champaign, Urbana, Illinois, USA; Washington University in St. Louis School of Medicine

**Keywords:** hydrogen sulfide, nitric oxide, nitroxyl, persulfide, reactive nitrogen species, reactive sulfur species, transcriptomics

## Abstract

*Staphylococcus aureus* is a commensal human pathogen and a major cause of nosocomial infections. As gaseous signaling molecules, endogenous hydrogen sulfide (H_2_S) and nitric oxide (NO·) protect *S. aureus* from antibiotic stress synergistically, which we propose involves the intermediacy of nitroxyl (HNO). Here, we examine the effect of exogenous sulfide and HNO on the transcriptome and the formation of low-molecular-weight (LMW) thiol persulfides of bacillithiol, cysteine, and coenzyme A as representative of reactive sulfur species (RSS) in wild-type and Δ*cstR* strains of *S. aureus*. CstR is a per- and polysulfide sensor that controls the expression of a sulfide oxidation and detoxification system. As anticipated, exogenous sulfide induces the *cst* operon but also indirectly represses much of the CymR regulon which controls cysteine metabolism. A zinc limitation response is also observed, linking sulfide homeostasis to zinc bioavailability. Cellular RSS levels impact the expression of a number of virulence factors, including the exotoxins, particularly apparent in the Δ*cstR* strain. HNO, like sulfide, induces the* cst* operon as well as other genes regulated by exogenous sulfide, a finding that is traced to a direct reaction of CstR with HNO and to an endogenous perturbation in cellular RSS, possibly originating from disassembly of Fe-S clusters. More broadly, HNO induces a transcriptomic response to Fe overload, Cu toxicity, and reactive oxygen species and reactive nitrogen species and shares similarity with the *sigB* regulon. This work reveals an H_2_S/NO· interplay in *S. aureus* that impacts transition metal homeostasis and virulence gene expression.

**IMPORTANCE** Hydrogen sulfide (H_2_S) is a toxic molecule and a recently described gasotransmitter in vertebrates whose function in bacteria is not well understood. In this work, we describe the transcriptomic response of the major human pathogen *Staphylococcus aureus* to quantified changes in levels of cellular organic reactive sulfur species, which are effector molecules involved in H_2_S signaling. We show that nitroxyl (HNO), a recently described signaling intermediate proposed to originate from the interplay of H_2_S and nitric oxide, also induces changes in cellular sulfur speciation and transition metal homeostasis, thus linking sulfide homeostasis to an adaptive response to antimicrobial reactive nitrogen species.

## Abstract (precis)

Hydrogen sulfide (H_2_S) is a toxic molecule and a recently described gasotransmitter in vertebrates whose function in bacteria is not well understood. In this work, we describe the transcriptomic response of the major human pathogen *Staphylococcus aureus* to quantified changes in levels of cellular organic reactive sulfur species, which are effector molecules involved in H_2_S signaling. We show that nitroxyl (HNO), a recently described signaling intermediate proposed to originate from the interplay of H_2_S and nitric oxide, also induces changes in cellular sulfur speciation and transition metal homeostasis, thus linking sulfide homeostasis to an adaptive response to antimicrobial reactive nitrogen species.

## INTRODUCTION

**FIG 1:**
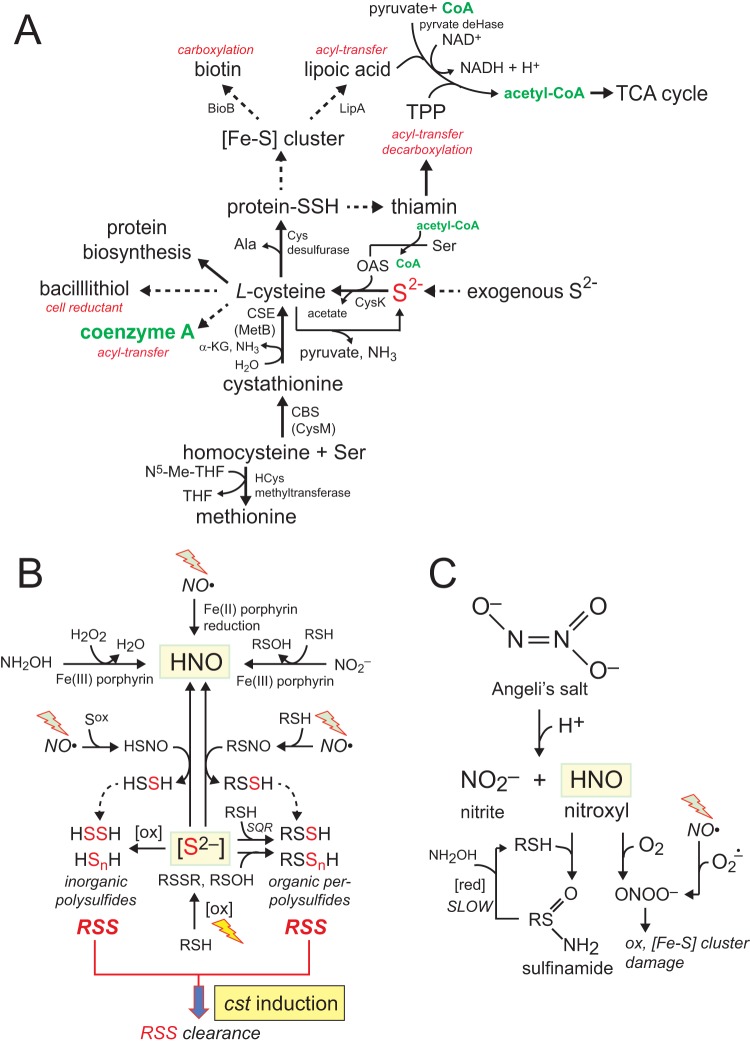
Sulfide (S^2−^) homeostasis and small-molecule chemistry that couples reactive sulfur species (RSS) and reactive nitrogen species (RNS). (A) Sulfide (S^2−^, HS^−^, and H_2_S) and *O*-acetyl-l-serine (OAS) are substrates for cysteine synthase (CysK) to form l-cysteine. l-Cysteine is the biosynthetic precursor to the major cellular reducing thiol in *S. aureus*, bacillithiol (BSH), and to coenzyme A (CoASH), which plays important roles in acyl-transfer reactions. l-Cysteine is also the precursor to other major sulfur-containing cofactors, including [Fe-S] clusters, biotin, lipoic acid, and thiamine pyrophosphate (TPP). Lipoic acid and TPP function in the pyruvate dehydrogenase (pyruvate deHase) complex (upper right), used to synthesize acetyl-CoA, which is fed into the tricarboxylic acid (TCA) cycle and other cellular processes. S^2–^ can be accumulate in cells either from exogenous sulfide sources or endogenously via the activity of two enzymes in a transsulfuration pathway, cystathionine β-synthase (CBS) and cystathionine γ-lyase (CSE), which converts homocysteine to l-cysteine. (B) Nitroxyl (HNO) defines an intersection of sulfide, LMW thiols (RSH), reactive sulfur species (RSS), reactive oxygen species (ox), and nitric oxide (NO·). Sulfide can lead to the accumulation of inorganic polysulfides (HS_n_H) and of organic persulfides (*n* = 1) and polysulfides (RSS_n_H), collectively termed reactive sulfur species (RSS), for which there is evidence in mammalian cells (16) and bacterial cells (26). These RSS are sensed by CstR (29), leading to the upregulation of a sulfide oxidation system that includes a canonical sulfide:quinone oxidoreductase (*SQR*) (26, 81). The RSS can also be derived from exogenous or endogenous NO·, which reacts with oxidized sulfur species (S^ox^, RS^ox^) to create the nitrosothiol thionitrous acid (HSNO) and organic nitrosothiols (RSNO), which in turn react with S^2–^ to make HNO (82-86). HNO can also be made via 1-electron reduction of NO· or other transformations (top of figure; note that not all possible reactions are shown [85]). (C) Angeli’s salt (AS) is an HNO donor (34) which, at pH values between 4 and 8, decomposes to HNO (pK_a_, 11.4) (87) and nitrite (NO_2_^−^). HNO further reacts with O_2_ to create the potent oxidant peroxynitrite (ONOO^−^), which in turn can react with S^2–^ of [Fe-S] clusters to give perthionitrite (SSNO^−^) (83), thus leading to the decomposition of protein-bound [Fe-S] clusters. SSNO^−^ is unstable in aqueous solution and may regenerate HNO (83). HNO is also a highly reactive electrophile at neutral pH that reacts with protein thiols to form sulfinamides in aqueous solution (56); this reaction is in competition with HNO dimerization to form N_2_O and H_2_O (66) (not shown).

Hydrogen sulfide (H_2_S) is both a toxic gas and a substrate for the biosynthesis of cysteine, an essential amino acid required for the synthesis of low-molecular-weight (LMW) thiols and the biogenesis of iron-sulfur (Fe-S) proteins (1, 2). Cysteine and H_2_S are also metabolic precursors for methionine and other sulfur-containing enzyme cofactors and thus significantly impact a wide range of metabolic activities in the cell (Fig. 1A). In bacteria, including the human pathogen *Staphylococcus aureus*, the cysteine pool is controlled by the global regulator CymR, which impacts sulfur metabolism, virulence gene expression, and survival of the organism both inside and outside the host (3–5). Sulfide (the term which we use here to refer collectively to H_2_S, HS^−^, and S^2−^) can be assimilated from exogenous sulfur sources or produced endogenously primarily by enzymes of the transsulfuration pathway, including cystathionine-β-synthase (CBS) and cystathionine-γ-lyase (CSE) (Fig. 1A). The toxicity of H_2_S coupled with its beneficial functions suggests a need for sulfide homeostasis, in which the cellular concentrations of sulfide and sulfide-derived reactive sulfur species are tightly regulated.

H_2_S freely passes through cell membranes, unlike its deprotonated conjugate bases (HS^−^ and S^2−^), and this property underscores recent suggestions that H_2_S, like nitric oxide (NO·) and carbon monoxide (CO), is a vertebrate gasotransmitter that functions as an endothelium-derived vasorelaxer (6, 7). Recent findings in mammalian cells suggest that H_2_S and NO· functionally interact to form nitroxyl (HNO), the one-electron reduced and protonated form of NO·, which controls the activity of the sensory chemoreceptor channel TRPA1 (8–10) (Fig. 1B). Likewise, in bacterial systems, H_2_S (11) and NO· (12) are reported to act synergistically to protect a number of bacterial strains against antibiotic stress, a finding that originates with the bacterially encoded nitric oxide synthases which are generally protective against immune oxidative attack (13, 14). *In vitro* experiments firmly establish that this cross talk between H_2_S and NO· leads to the formation of HNO as a primary species, as well as of thiol persulfides (RSS^−^) and organic (RSS_n_^−^) and inorganic (HS_n_^−^) polysulfides (*n* > 1) as bioactive products (Fig. 1B) (15). These per- and polysulfide species, collectively termed reactive sulfur species (RSS), are reportedly maintained in relatively high concentrations (0.01 to 0.1 mM) in mammalian cells and are proposed to function as true small-molecule signaling species in an H_2_S signaling pathway (16).

Although our understanding of the biological impact of HNO is not yet complete, HNO appears to be characterized by a chemical reactivity and biogenesis profile that is distinct from that of NO·. It is thought that the reduction of NO· by specific Fe(II)-heme and high-spin Fe(II) or Mn(II) complexes makes them potential sources of endogenous HNO in living cells (17). HNO is a highly reactive and short-lived molecule that self-quenches via dimer formation to yield nitrous oxide (N_2_O) (18). However, prior to self-quenching, this potent electrophile is capable of reacting with nucleophilic thiolate groups to form sulfinamides (Fig. 1C) (19, 20). In addition, while HNO reacts only slowly with molecular oxygen at neutral pH to generate peroxynitrite (ONOO^−^) (21), ONOO^–^ is formed from NO· and superoxide anion at diffusion-controlled rates (Fig. 1C) (22, 23). Thus, the presence of multiple reactive nitrogen species (RNS) and reactive oxygen species (ROS) at the host-pathogen interface may lead to the formation of ONOO^−^, which is a strong oxidant that causes lipid peroxidation, nitration of aromatic residues, thiol oxidation, and disassembly of Fe-S clusters (24) (Fig. 1C).

There is emerging evidence that endogenously produced sulfide and a pool of RSS and perhaps downstream products (Fig. 1B) protect cells against the effects of ROS (16, 25, 26). Indeed, proteome *S*-sulfhydration might constitute a reservoir of persulfidated cysteines while driving up- and downregulation of metabolic pathways to provide protection against oxidative stress (16, 25, 27). In this model, cells must be capable of both biosynthesis and clearance of RSS to maintain sulfide homeostasis. In previous work, we discovered and characterized a novel per- and polysulfide-sensing dithiol-containing repressor from the major nosocomial pathogen *S. aureus*, named CstR (for "CsoR-like sulfurtransferase repressor") (28, 29). CstR represses transcription of *cstA*, *cstB*, and *sqr*, which together encode an H_2_S oxidation system (S^2–^ to thiosulfate, S_2_O_3_^2−^) (26, 30, 31). Sulfide-responsive and RSS-sensing repressors that are functionally analogous to CstR and yet are structurally unrelated are also found and have recently been characterized in Gram-negative bacteria, suggesting that sulfide homeostasis may be more widespread than previously anticipated (32, 33).

In this work, we employed transcriptomic and organic persulfide metabolite profiling approaches to investigate the cellular response of *S. aureus* to sodium sulfide and a commonly used HNO donor, Angeli’s salt (AS) (34) (Fig. 1C), added exogenously to cells. We found that sulfide treatment induces the *cst* operon as expected (29) and strongly downregulates a subset of genes regulated by CymR, the master regulator of cysteine biosynthesis (3, 5). We further show that AS treatment bears some similarity to sulfide stress, a finding directly attributable to HNO and not nitrite (NO_2_^−^) (29). Both sulfide stress and HNO also result in a significant zinc starvation response, while the Δ*cstR* strain, characterized by lower levels of RSS, shows significant repression of the expression of immunomodulators and superantigen toxins, some of which are controlled by the global virulence regulator MgrA (35). The implications of these findings for the biological pathway(s) influenced by sulfide/RSS signaling and H_2_S/HNO interplay in virulence gene expression and transition metal bioavailability are discussed.

## RESULTS AND DISCUSSION

### Transcriptomic analysis of the effects of exogenous sulfide stress and a Δ*cstR* strain.

Previous work showed that addition of sodium sulfide to *S. aureus* cells aerobically grown to the early log phase resulted in the transient induction of the *cst* operon and massive accumulation of organic LMW persulfides in cells (26, 29). In contrast, unregulated expression of genes in a *cst* operon in a Δ*cstR* stain reduced the levels of these cellular RSS below those seen with wild-type cells (see below). In order to understand the impact of cellular sulfide and RSS concentrations on global gene expression, we carried out a transcriptomic analysis of mid-log *S. aureus* strain Newman treated with 0.2 mM NaHS for 10 min versus an untreated Δ*cstR* strain and of both relative to untreated wild-type cells (see Table S1A and Fig. S1 in the supplemental material). Totals of 38 genes and 37 genes, respectively, exhibited an increase or decrease in expression of more than 3.0-fold (1 standard deviation from the mean induction level; see Table S1A) in sulfide-treated cells and in untreated wild-type cells (Tables 1 and 2, respectively). These genes included *cstA*, *cstB*, and *sqr*, all direct targets of CstR regulation (29), as well as a number of enzymes and regulators involved in sugar (*glpF*, *marR*, *gapB*, *scrR*, *gntK*, and *gntR*) and amino acid (*putA*) metabolism.

10.1128/mSphere.00082-17.1 FIG S1  qRT-PCR validation of the RNAseq results. (A) Subset of the genes (expressed as *S. aureus* strain Newman locus tags, NWMN_wxyz) induced (compare to Table 1) or repressed (Table 2) upon treatment with 0.2 mM Na_2_S at 10 min posttreatment relative to unstressed cells. (B) Subset of genes induced (compare to Table 3) and repressed (Table 4) in the unstressed, early log-phase Δ*cstR* strain relative to the isogenic wild-type strain cultured under the same conditions. (C) Subset of the genes induced (compare to Table 1) and repressed (Table 2) upon treatment with 0.2 mM AS at 10 min posttreatment relative to unstressed cells. Download FIG S1, EPS file, 1.1 MB. Copyright © 2017 Peng et al. 2017 Peng et al. This content is distributed under the terms of the Creative Commons Attribution 4.0 International license.

10.1128/mSphere.00082-17.10 TABLE S1  Complete transcriptomic data and sequences of qRT-PCR primers used in this study. (A) Excel file listing all genes whose expression is altered in sulfide-treated cells (sheets 1 to 2, corresponding to Fig. 4 and Tables 1 and 2, main text), in the Δ*cstR* strain (sheets 3 to 4, corresponding to Fig. 4 and Tables 3 and 4, main text), in AS-treated cells (sheets 5 to 6, corresponding to Fig. 6 and Tables 1 and 2, main text), and in CP-treated cells (sheets 7 to 8, corresponding to Fig. 3, main text) that meet the minimal acceptance criteria (2-fold change in expression; adjusted *P* value of ≤0.05). (B) A list of all the primers used in the qRT-PCR experiments. Download TABLE S1, XLSX file, 0.1 MB. Copyright © 2017 Peng et al. 2017 Peng et al. This content is distributed under the terms of the Creative Commons Attribution 4.0 International license.

**FIG 2:**
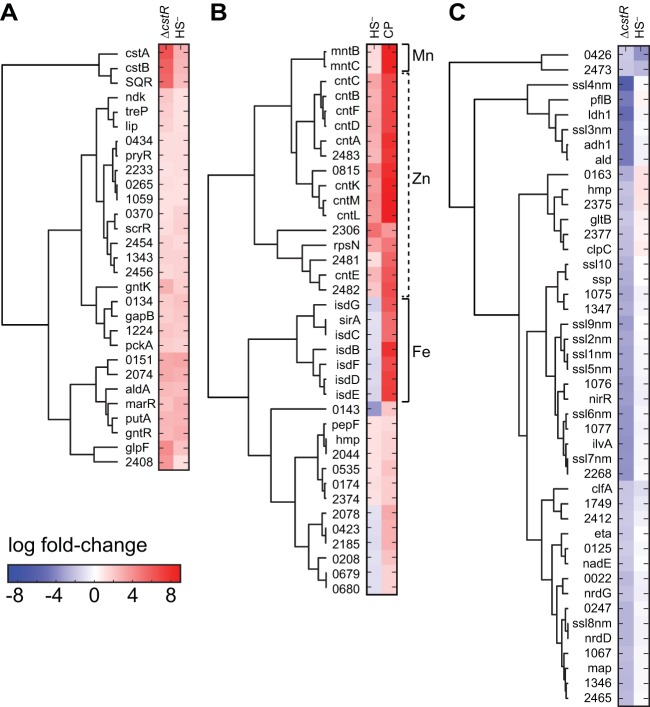
Clustering analysis and RNAseq transcriptomic analysis of *Staphylococcus aureus* strain Newman. Genes that change expression significantly in pairwise comparisons of results of sulfide (HS^−^) treatment versus the Δ*cstR* strain (upregulated genes only) (A) or of sulfide treatment versus CP treatment (all genes) (B) are indicated. (C) A list of genes in the Δ*cstR* strain (Table 4) that are significantly downregulated compared to their expression under sulfide stress conditions. Genes are clustered according to similarities in changes in expression in each pair of experiments.

A subset of genes that were upregulated in both the sulfide-stressed wild-type and Δ*cstR* cultures (Table 3) relative to untreated wild-type *S. aureus* cultures included *aldA* (NWMN_0113), encoding an uncharacterized aldehyde dehydrogenase, and hypothetical operons beginning with NWMN_0134 (NWMN_0134 to NWMN_0137) and NWMN_0151 (NWMN_0151 to NWMN_0154), the latter of which is associated with changes in carbohydrate metabolism and uptake in other *Staphylococcus* strains. Although some of these sulfide-inducible genes are also induced in the Δ*cstR* strain, none would appear to be direct targets of CstR regulation since they lack clearly identifiable *cstR* operators upstream (28) in the immediately adjacent intergenic regions, a finding supported by our clustering analysis (Fig. 2A, bottom). These data, taken collectively, suggest that CstR likely directly regulates the expression of a single operon, *cst*, with other genes induced by sulfide treatment likely indirectly influenced by metabolic changes in the cell, which includes RSS.

Comparing this list of sulfide-induced genes relative to the untreated control wild-type strain with results of other transcriptomics experiments using the *S. aureus* transcript regulatory network analysis tool (SATRAT) (36) reveals that a majority of these genes were oppositely (down)regulated by oxidative stress induced by short-time-scale exogenously added hydrogen peroxide (Fig. S2A and C) (37) and hypochlorous acid. In addition, the ratio of reduced to oxidized bacillithiol (BSH), one of the major LMW thiols in *S. aureus* (see below), increased upon sulfide treatment relative to untreated cells (Fig. S3), which is opposite what is expected under conditions of oxidative stress. These data collectively reveal that the transcriptomic response induced by changes in cellular RSS is opposite that induced by this potent oxidant (Table S1A). Thus, RSS and ROS transcriptional programs operate independently in the cell, consistent with what is known about the inducer specificity of CstR relative to that of PerR, the major ROS-sensing repressor in *S. aureus* (29, 38).

10.1128/mSphere.00082-17.2 FIG S2  Analysis of all transcriptomic (RNAseq) changes in the sulfide-stressed (HS^−^) and AS-treated cells that showed significant changes in other stress-treated cells. These stresses included oxidative stress (ROS) (10 mM H_2_O_2_) (A), nitrosative stress (RNS) (150 μM GSNO [*S*-nitrosoglutathione]) (B), and combined ROS/RNS stress (10 mM H_2_O_2_ plus 150 μM GSNO) (C) (37). The cutoff is 2-fold change. Gene names are shown (where known) on the right. Note that only genes that are significantly regulated by either sulfide or AS stress or both sulfide and AS stress and under other stress conditions are shown. (A) The 90 genes shown represent the 45% of the transcriptome that is affected by acute-phase ROS, corresponding to 56% of upregulated and 18% of downregulated genes that change in the same direction. (B) The 43 genes shown represent the 41% of the transcriptome that is affected by GSNO, corresponding to 37% of upregulated and 19% of downregulated genes that change in the same direction. (C) The 123 genes shown represent the 43% of the transcriptome that is affected by the combined ROS/RNS stress, corresponding to 33% of upregulated and 36% of downregulated genes that change in the same direction. Download FIG S2, EPS file, 2.6 MB. Copyright © 2017 Peng et al. 2017 Peng et al. This content is distributed under the terms of the Creative Commons Attribution 4.0 International license.

10.1128/mSphere.00082-17.3 FIG S3  The ratio of reduced to oxidized bacillithiol (BSH/BSSB) in the sulfide-stressed (HS^–^) and HNO-treated cells compared to untreated cells. Sulfide treatment increased BSH/BSSB levels significantly, but the ratio remained unchanged under AS treatment. The experiments were done in triplicate, with standard deviations shown as error bars. Statistical significance relative to untreated cells was established by using a paired *t* test (***, *P* < 0.001; n.s., no significant change). Download FIG S3, EPS file, 0.6 MB. Copyright © 2017 Peng et al. 2017 Peng et al. This content is distributed under the terms of the Creative Commons Attribution 4.0 International license.

### Exogenous sulfide induces a zinc limitation transcriptomic response.

**FIG 3:**
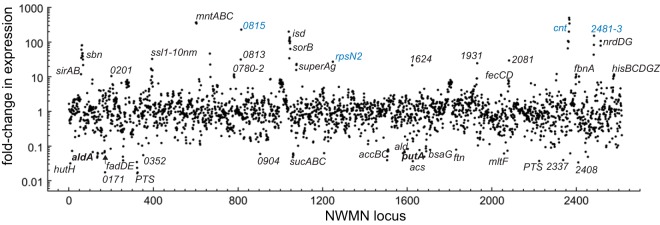
RNAseq transcriptomic analysis of wild-type *Staphylococcus aureus* strain Newman treated with calprotectin (CP). The fold change in expression for each locus tag is indicated (see Table S1A for a complete list of genes induced ≥2.0-fold and with adjusted *P* values of ≤0.05). Gene names are indicated where known; otherwise, the locus identifier (NWMN_wxyz [where "wxyz" represents the locus number]) is indicated. Names in blue are genes also induced by sulfide (Fig. 4).

In addition to the transcriptomic response to exogenous sulfide treatment described above, exogenous sulfide stress also induces a pronounced zinc limitation response in *S. aureus* that is similar to that mediated by the antimicrobial protein calprotectin (CP) (Fig. 2B and Fig. 3). For example, sulfide treatment induces strong upregulation of the zinc uptake repressor (Zur) regulon (39) (Table 1; Table S1A), while downregulating the expression of *czrAB* (*zntRA* [40]), encoding the Zn efflux regulator and cation diffusion facilitator transporter (Table 2) (41, 42). Sulfide treatment also leads to upregulation of *mntABC*, controlled by the Mn-sensing repressor MntR (43), but no measurable Fe limitation response, in contrast to CP treatment (Fig. 2B). Genes that are upregulated by both exogenous sulfide treatment and CP treatment and linked to transition metal homeostasis include NWMN_2481, encoding a putative COG0523 G3E family GTPase linked to zinc homeostasis in other organisms (44, 45); two genes encoding uncharacterized pyridine nucleotide disulfide oxidoreductases (NWMN_0815 and NWMN_2370); *rpmG2* and *rpsN2*, encoding two non-zinc-containing paralogs of ribosomal proteins (46); the *adcA* gene encoding a zinc-binding lipoprotein (NWMN_2306); and the entire *cnt* gene cluster, *cntA* to *cntF* (*cntA-F*) (Table 1) (Fig. 2B and Fig. 3). *cntA-F* is reported to encode a Co/Ni uptake system that is expressed upon metal limitation (47), while *cntKLM* encodes the biosynthetic machinery that produces a broad-spectrum nicotianamine-like metallophore that is capable of scavenging metals from the environment (48).

**TABLE 1:** List of genes upregulated in response to NaHS stress (≥3-fold) or by Angeli’s salt (≥10-fold)

Locus tag (Newman)	Locus tag (N315)	Gene name	Fold induction (S^2−^)	Fold induction (Angeli’s salt)^a^	Function	Plus CP treatment^b^ (fold increase)?	Δ*cstR* regulation?
NWMN_0026	SA0080	*tauE*		5.5	Putative sulfonate/thiosulfate (TS) efflux		Yes
NWMN_0027	SA0082	*cstA*	6.1	11.5	Multidomain sulfurtransferase^a^		Yes
NWMN_0028	SA0083	*cstB*	5.8	7.5	Persulfide dioxygenase-sulfur transferase^b^		Yes
NWMN_0029	SA0084	*sqr*	4.9	6.4	Sulfide-quinone reductase^b^		Yes
NWMN_0047	SA0098			15.0	Hypothetical		
NWMN_0048	SA0099			14.1	Hypothetical		
NWMN_0071	SA0122		2.0^c^	12.2	Acetoin reductase		
NWMN_0113	SA0162	*aldA*	4.8	56.6	Aldehyde dehydrogenase-like		Yes
NWMN_0134	SA0184		4.4	7.2	Hypothetical (NWMN_0134–0137; carbohydrate metabolism)		
NWMN_0151	SA0206		8.6	7.5	Hypothetical (NWMN_0151–0154 operon)		Yes
NWMN_0162	SA0218	*pflB*		8.1	Formate acetyltransferase		
NWMN_0163	SA0219			19.1	Formate-lyase activating enzyme		
NWMN_0173	SA0229		2.2	7.5	Hypothetical		
NWMN_0174	SA0230		2.1	16.0	Hypothetical	Yes (3.4)	
NWMN_0175	SA0231		2.2	20.7	Flavohemoprotein	Yes (2.9)	
NWMN_0330	SA0326			8.0	Glyoxalase-like protein		
NWMN_0331	SA0327			9.2	Luciferase-like monooxygenase		
NWMN_0332	SA0328			9.2	NADH-dependent flavin mononucleotide (FMN) reductase (like NWMN_2421)		
NWMN_0371	SA0365			5.7*	Alkyl hydroperoxide reductase subunit F		
NWMN_0372	SA0366			5.8*	Alkyl hydroperoxide reductase subunit C		
NWMN_0373	SA0367			13.7	Nitroreductase family protein		
NWMN_0410	SA0396	*lpl7nm*		4.0	Tandem lipoprotein (NWMN_0410–0411 operon)		
NWMN_0417	SA0410	*cobW*	7.2		Putative COG0523 GTPase		
NWMN_0418	SA0411	*ndhF*	3.6	6.6	Putative NADH dehydrogenase subunit 5		
NWMN_0484	SA0480	*ctsR*		16.3	Transcriptional regulator CtsR (stress induced)		
NWMN_0485	SA0481			20.6	UvrB/UvrC motif-containing protein		
NWMN_0486	SA0482			16.1	ATP:guanido phosphotransferase		
NWMN_0487	SA0483	*clpC*		12.7	ClpC protease, ATP-binding subunit		
NWMN_0587	SA0572			15.5	Hypothetical		
NWMN_0669	SA0655	*fruA*		14.2	Fructose permease (NWMN_0667–0669, *frc* operon)		
NWMN_0815	SA0806		17.5	8.4	NADH-dependent pyridine nucleotide disulfide oxidoreductase (short-chain dehydrogenase/reductase [SDR])	Yes (229)	
NWMN_0826	SA0817			13.1	NADH-dependent flavin oxidoreductase		
NWMN_0845	SA0835			28.2	ClpB protease, ATP binding subunit		
NWMN_0900			3.2		Hypothetical (57 residues)		
NWMN_1084			3.5	−4.1	Anti-protein (44 residues)		
NWMN_1207	SA1140	*glpF*	4.3	2.1	Glycerol uptake facilitator protein		Yes
NWMN_1224			3.4		Hypothetical (82 residues)		
NWMN_1246	SA1170	*katA*	2.5	26.8	Catalase		
NWMN_1247		*rpmG2*	6.6	3.6	Non-Zn-containing paralog *rpmG*	Yes (2.0)	
NWMN_1248	SA1171	*rpsN2*	9.1	4.2	Non-Zn-containing paralog *rpsN* (S14)	Yes (27.3)	
NWMN_1415	SA1339	*marR*	6.3	4.5	Putative PurR/LacI family repressor		Yes
NWMN_1483	SA1409	*dnaJ*		9.1	Molecular chaperone DnaK (hsp70)		
NWMN_1484	SA1410	*grpE*		8.9	Heat shock protein GrpE		
NWMN_1485	SA1411	*hrcA*		11.1	Heat-inducible transcriptional repressor		
NWMN_1580	SA1510	*gapB*	3.7	3.9	glyceraldehyde-3-phosphate dehydrogenase 2		
NWMN_1658	SA1585	*putA*	5.8	17.1	Proline dehydrogenase		Yes
NWMN_1709		*bsaG*		15.2	ABC transporter protein: lantibiotic		
NWMN_1710		*bsaE*		9.5	ABC transporter protein: lantibiotic		
NWMN_1711		*bsaF*		8.6	ABC transporter protein: lantibiotic		
NWMN_1929	SA1814	*dapE*		9.5	Succinyl-diaminopimelate desuccinylase (dinuclear)		
NWMN_1949	SA1847	*scrR*	3.1	4.0	Sucrose operon repressor		
NWMN_2026	SA1924			11.3	Aldehyde dehydrogenase family		
NWMN_2043	SA1941			25.8	Dps; nonheme Fe-containing ferritin	Yes (3.0)	
NWMN_2044	SA1942			8.0	Hypothetical; predicted disulfide oxidoreductase	Yes (2.9)	
NWMN_2048	SA1946			19.1	Hypothetical		
NWMN_2059	SA1962	*mtlA*		14.7	Mannitol-specific IIA component (2057–2060 operon)		
NWMN_2060	SA1963	*mtlD*		15.4	Mannitol-1-phosphate 5-dehydrogenase		
NWMN_2074	SAS074		6.3	5.9	Hypothetical		Yes
NWMN_2086	SA1984			15.1	Alkaline shock protein 23		
NWMN_2087	SA1985			18.3	Hypothetical (79 residues; COG5547)		
NWMN_2088	SA1986			11.8	Hypothetical		
NWMN_2091	SA1989			10.9	Hypothetical (quinone oxidoreductase)		
NWMN_2109	SA2006			12.2	Truncated MHC class II analog protein		
NWMN_2180	SA2075			13.3	Formate dehydrogenase accessory protein		
NWMN_2209	SA2101			10.7	Hypothetical		
NWMN_2210	SA2102			5.1^5^	Formate dehydrogenase-like		
NWMN_2229	SA2119			15.2	Hypothetical		
NWMN_2273	SA2161			13.6	Acetyltransferase, GNAT family protein		
NWMN_2274	SA2162			13.6	Pyridine nucleotide-disulfide oxidoreductase (TrxB-like)	Yes (3.4)	
NWMN_2282	SA2170			12.3	Hypothetical		
NWMN_2306	SA2184		32.0		Zinc-binding lipoprotein, AdcA-like	Yes (12.1)	
NWMN_2359	SA2250	*cntE*	5.6		Major facilitator superfamily (MFS)	Yes (66.1)	
NWMN_2360	SA2251	*cntF*	7.2		ABC transporter; cobalt-nickel	Yes (108)	
NWMN_2361	SA2252	*cntD*	7.9		ABC transporter; cobalt-nickel	Yes (107)	
NWMN_2362	SA2253	*cntC*	9.0		ABC transporter; cobalt-nickel	Yes (104)	
NWMN_2363	SA2254	*cntB*	6.5	3.7	ABC transporter; cobalt-nickel	Yes (101)	
NWMN_2364	SA2255	*cntA*	6.6	5.0	ABC transporter; cobalt-nickel	Yes (199)	
NWMN_2365	SA2256	*cntM*	13.8	10.0	Hypothetical (NWMN_2367–2365 operon)	Yes (504)	
NWMN_2366	SA2257	*cntL*	12.1	15.7	Hypothetical, epimerase-like	Yes (445)	
NWMN_2367	SA2258	*cntK*	11.2	14.2	Hypothetical	Yes (346)	
NWMN_2368	SA2259			8.9	Hypothetical		
NWMN_2369	SA2260			15.1	Short-chain dehydrogenase		
NWMN_2402	SA2294	*gntK*	3.1	6.0	Gluconate kinase		Yes
NWMN_2403	SA2295	*gntR*	6.9	5.5	Gluconate operon repressor		Yes
NWMN_2414	SA2304	*fbp*	2.1	12.2	Fructose-1,6-bisphosphatase		
NWMN_2434	SA2323			5.0	Hypothetical		
NWMN_2435	SA2324			11.0	Hypothetical		
NWMN_2436	SA2325			10.5	Hypothetical		
NWMN_2456	SA2343		3.0		Hypothetical (63 residues)		
NWMN_2457	SA2344	*copA*		68.3	Cu(I)-specific P-type ATPase efflux transporter^6^		
NWMN_2458	SA2345	*copZ*		26.1	Cu(I) chaperone^6^		
NWMN_2461	SA2348	*ctrM*		5.6	Squalene synthase		
NWMN_2462	SA2349	*ctrN*		9.0	Squalene synthase		
NWMN_2463	SA2350			13.6	Glycosyl transferase, group 2 family protein^7^		
NWMN_2464	SA2351	*crtI*		4.6	Phytoene dehydrogenase		
NWMN_2479	SA2366			6.8	Amidohydrolase family protein		
NWMN_2480	SA2367			11.5	α/β hydrolase family protein		
NWMN_2481	SA2368		2.4^d^	1.7	Putative COG0523 GTPase	Yes (43.2)	
NWMN_2482	SA2369		4.0	2.6	Hypothetical	Yes (83.1)	
NWMN_2483	SA2370		5.5	5.0	FAD-dependent pyridine nucleotide disulfide oxidoreductase	Yes (152)	

a Fold induction is shown where S^2−^ induction is ≥3.0-fold.

b See Table S1A.

c ≥2-fold induction with NaHS in HNO induction is statistically significant (see Table S1A; adjusted *P* value, ≤0.05).

d Below indicated cutoff values but part of an operon harboring genes above the cutoff value.

**TABLE 2:** List of genes downregulated in response to NaHS stress (≥3-fold) or by Angeli’s salt (≥5-fold)

Locus tag (Newman)	Locus tag (N315)	Gene name	Fold repression (S^2−^)	Fold repression (Angeli’s salt)^a^	Function	CymR regulon?^b^	Δ*cstR* regulation?
NWMN_0115	SA0165		16.9	4.2	Hypothetical	+	
NWMN_0116	SA0166		34.0	5.7	ABC transporter: putative TauB (sulfonate)	+	
NWMN_0117	SA0167		11.5	5.1	ABC transporter: putative TauA	+	
NWMN_0118	SA0168		13.0	6.6	ABC transporter: putative TauC	+	
NWMN_0119	SA0169		13.7	3.6	Putative CiaA: NAD-dependent formate dehydrogenase	+	
NWMN_0143	SA0198		11.9	3.1	ABC transporter: peptide (ATPase)^c^	+	−/+ down^d^
NWMN_0144	SA0199		9.4	11.1	ABC transporter: peptide (permease)^c^	−	
NWMN_0145	SA0200		11.8	9.4	ABC transporter: peptide (permease)^c^	−	−/+ down^d^
NWMN_0146	SA0201	*rlp*	6.0	4.3	Putative RGD-containing lipoprotein	−	
NWMN_0374	SA0368	*tcyP*	7.5	4.4	Putative cystine transporter homolog	+	
NWMN_0391	SA0385	*ssl4nm*		5.1	Superantigen-like protein 4	−	Down
NWMN_0400		*ssl11nm*	3.3	5.4	Superantigen-like protein 11	−	−/+ down^d^
NWMN_0401			3.4	7.1	Hypothetical	−	−/+ down^d^
NWMN_0423	SA0417		2.3^e^	5.4	Sodium-dependent symporter	−	−/+ up^d^
NWMN_0424	SA0418	*cysM*	5.7		Cystathionine-β-synthase (CBS)	+	
NWMN_0425	SA0419	*metB*	8.5		Cystathionine-γ-lyase (CSE)	+	
NWMN_0426	SA0420		13.0	11.6	ABC transporter: peptide (ATPase)	−	−/+ down^d^
NWMN_0427	SA0421		13.9	11.9	ABC transporter: peptide (permease)	−	−/+ down^d^
NWMN_0428	SA0422		9.9	4.1	ABC transporter: peptide (substrate binding)	−	−/+ down^d^
NWMN_0475	SA0471	*cysK*	10.4		Cysteine synthase (OAS + H_2_S → acetate + Cys)	+	
NWMN_0557	SA0551		7.5	Up 4.6	Putative pyridine nucleotide disulfide oxidoreductase	−	
NWMN_0558	SA0552		16.6		Rrf2 family repressor (related to *B. subtilis* SaiR)	−	
NWMN_0655	SA0641	*mgrA*		5.1	MarR family repressor MgrA	−	
NWMN_0757			2.3^e^	28.8	Secreted von Willebrand factor-binding protein	−	−/+ down^d^
NWMN_0759			3.0		Hypothetical	−	−/+ down^d^
NWMN_0766	SA0751		4.2		Hypothetical	−	
NWMN_0813	SA0804			5.8	Sodium/proton antiporter family protein	−	
NWMN_0831	SA0821	*argH*		3.0	Argininosuccinate lyase (Arg biosynthesis)	−	−/+ down^d^
NWMN_0832	SA0822	*argG*		6.2	Argininosuccinate synthase (Arg biosynthesis)	−	−/+ down^d^
NWMN_0907	SA0890			6.5	Hypothetical	−	
NWMN_1047	SA0983	*isdG*	3.1^f^		Heme-degrading monooxygenase	−	
NWMN_1066	SA1000			5.1	Hypothetical (fibrinogen binding)	−	
NWMN_1068	SA1002			5.6	Hypothetical	−	
NWMN_1069	SA1003			3.4	Hypothetical (fibrinogen binding)	−	
NWMN_1070	SA1004			6.9	Hypothetical (fibrinogen binding)	−	
NWMN_1352	SA1275			5.0	Hypothetical	−	
NWMN_1749	SA1674	*tcyC*	1.8^e^	7.1	ABC transporter: cystine (ATPase)^e^	+	Down
NWMN_1750	SA1675	*tycB*	1.6^e^	6.7	ABC transporter: cystine (permease)^e^	+	Down
NWMN_1751	SA1676	*tycA*	1.2^e^	4.8	ABC transporter: cystine (cystine binding)^e^	+	
NWMN_1877	SA1755	*chp*		32.7	Chemotaxis-inhibiting protein	−	
NWMN_1951	SA1849		9.8	4.4	TusA-like (SirA/YedF/YeeD) protein	+	
NWMN_1952	SA1850		14.2	5.0	Putative thiosulfate (TS) importer	+	
NWMN_2049	SA1949	*czrA*	15.9		Zinc-specific repressor (ArsR family)	−	
NWMN_2050	SA1950	*czrB*	14.0		Zinc cation diffusion facilitator (CDF) transporter	−	
NWMN_2075	SA1976			11.8	Hypothetical	−	
NWMN_2186	SA2080	*ydbM*	9.3		Putative CiaA; acyl (butyryl)-CoA dehydrogenase	+	
NWMN_2199	SA2093		2.1^e^	4.2	Secretory antigen precursor SsaA	−	
NWMN_2200	SA2094	*nhaC*		8.4	Sodium/proton antiporter family, NhaC	−	
NWMN_2201	SA2095		2.1^e^	9.5	Dehydrogenase family protein	−	
NWMN_2203				6.5	Secretory antigen precursor SsaA	−	
NWMN_2265	SA2153			5.5	Hypothetical	−	
NWMN_2311	SA2200		6.1		ABC transporter: amino acid (ATPase)	−	
NWMN_2312	SA2201		5.3		ABC transporter: amino acid (permease)	−	
NWMN_2313	SA2202		10.1		ABC transporter: amino acid (substrate binding)	−	
NWMN_2317	SA2206	*sbi*		5.0	Immunoglobulin G-binding protein	−	
NWMN_2470	SA2357			5.2	Hypothetical (regulatory protein)	−	
NWMN_2472	SA2359		7.0		Hypothetical (just upstream of *dtr*)	−	
NWMN_2473	SA2360		5.4		Hypothetical (just upstream of *dtr*)	−	−/+ down^d^
NWMN_2577	SA2471	*hisG*		7.9	ATP phosphoribosyltransferase C subunit (His biosynthesis)	−	
NWMN_2578	SA2472	*hisZ*		6.0	ATP phosphoribosyltransferase R subunit (His biosynthesis)	−	

a Fold induction shown if S^2−^ induction is ≥3.0-fold.

b CymR regulon as determined previously on TSB plus 2.0 mM cysteine (3).

c Proposed to be involved in glutathione (GSH) assimilation and degradation (3).

d Approximately 2-fold repressed (downregulated [down]) or activated (upregulated [up]) in the Δ*cstR* strain (see Tables 3 and 4 for a list of genes differentially expressed in the Δ*cstR* strain), with adjusted *P* value of ≤0.05.

e At least 2-fold induction with NaHS (see Table S1A; adjusted *P* value of ≤0.05) in HNO induction is significant.

f The entire *isd* operon is modestly (≥2-fold) repressed (see Table S1A for a complete listing of these genes).

Induction of a zinc starvation response by application of exogenously added sulfide can be traced to the ability of bisulfide (HS^−^) salts to form stable coordination complexes with transition metals, leading to their precipitation from solution and making them nonbioavailable (49). Analysis of the growth medium before and after addition of 0.2 mM Na_2_S reveals an approximately 10-fold decrease in total Zn levels, with relatively smaller reductions in Cu(II) and Ni and no change in Mn and Fe (Fig. S4A). This suggests that increased levels of endogenous sulfide in the cytoplasm may lead to a reduction in the bioavailability of intracellular transition metals, particularly Zn, by chelation.

10.1128/mSphere.00082-17.4 FIG S4  Metal analysis of the HHWm growth medium before and after treatment with 0.2 mM NaHS (A) and 0.2 mM AS (B). ICP-MS was used to quantify the concentrations of transition metal ions in the HHWm growth medium (gray bars), HHWm plus chloramphenicol plus 0.2 mM thiosulfate (TS) as the sole sulfur source (red bars), or HHWm plus chloramphenicol plus 0.2 mM thiosulfate plus 0.2 mM NaHS or AS (light blue bars). Sulfide treatment led to a significant decrease in the level of bioavailable (soluble) Zn (15.2 µM to 1.2 µM), with correspondingly small changes in the levels of Cu (approximately 2-fold) and Ni (less than 2-fold). AS treatment led to a significant decrease in the level of bioavailable (soluble) Zn (18.2 µM to 5.9 µM), with a correspondingly small change in the level of Ni (1.8-fold). Download FIG S4, EPS file, 2.7 MB. Copyright © 2017 Peng et al. 2017 Peng et al. This content is distributed under the terms of the Creative Commons Attribution 4.0 International license.

### Part of the CymR regulon is repressed by exogenous sulfide treatment.

**FIG 4:**
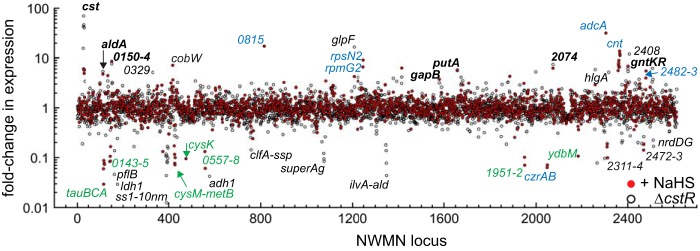
RNAseq transcriptomic analysis of *Staphylococcus aureus* strain Newman. Cells were either treated with 0.2 mM NaHS (red filled circles) or left untreated (Δ*cstR* strain) (black open symbols); data are expressed relative to the untreated wild-type strain results. The fold change in expression for each locus tag (NWMN_wxyz) is indicated (see Tables 1 to 4 for partial lists of these genes and Table S1A for a complete list). Gene names are indicated where known. Black bold type is used to represent genes that change expression in sulfide-treated cells, in Angeli’s salt (AS; nitroxyl)-treated cells, and in the Δ*cstR* strain; light blue type is used to represent genes observed to change in the calprotectin-treated samples; green type is used to represent genes of the CymR regulon (3).

**FIG 5:**
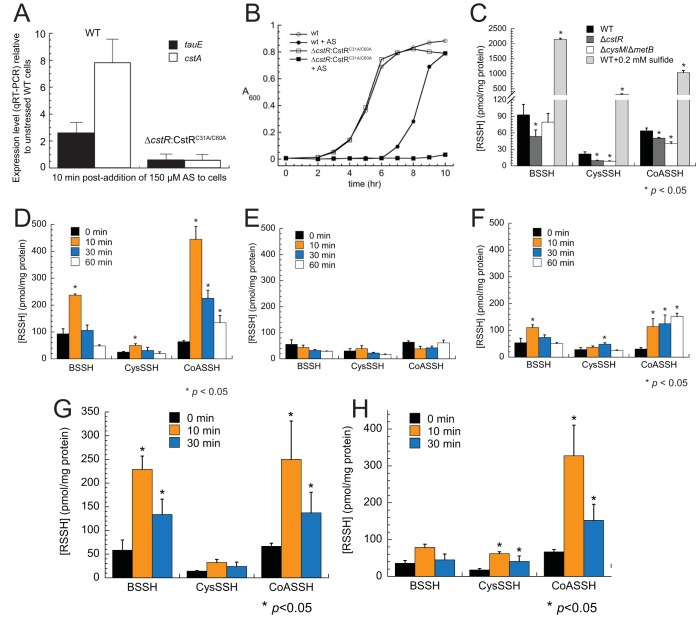
Angeli’s salt (AS) induces expression of the *cst* operon in a CstR-dependent manner and gives rise to a transient increase in endogenous LMW thiol persulfide levels, attributable to HNO. (A and B) AS induces *cst* operon expression as measured by qRT-PCR (A) and gives rise to a measurable growth phenotype when cells express an inactive CstR, C31A/C60A CstR (29) (B). WT, wild type. (C) LMW persulfide levels can be manipulated by genetic background and exogenous sulfide exposure. (D and E) AS (D) causes increased levels of LMW persulfides under aerobic conditions but nitrite (E) does not, indicating that HNO induces a transient increase in cellular RSS in *S. aureus*. (F) Levels of LMW persulfides in a Δ*cysM* Δ*metB* strain were also transiently increased by AS. (G and H) Both AS treatment under microaerophilic conditions (G) and ONOO^–^ treatment under aerobic conditions (H) cause an increase in the cellular accumulation of LMW persulfides. Error bars represent standard deviations of results of triplicate biological experiments, with statistical significance relative to the results seen with untreated wild-type cells (C) or to the results seen with each of the endogenous LMW persulfides at 0 min (D to H) established using paired *t* tests (*, *P* ≤ 0.05). Note that the quantitations of [RSSH] for *t* = 0 min in panels C, D, E, and H differ slightly from one another, reflecting the culture-to-culture variability of the measurements. Over all 12 replicates, [BSSH] = 77.1 ± 10.5 pmol/mg protein, [CysSSH] = 22.9 ± 2.6 pmol/mg protein, and [CoASSH] = 64.3 ± 3.0 pmol/mg protein, values fully consistent with previously published findings (26).

The suite of genes that are strongly (≥3.0-fold) repressed under conditions of exogenous sulfide stress are largely limited to a subset of CymR-regulated genes (5, 50), defined by differential expression in a *cymR* mutant *S. aureus* strain, strain N315 (Table 2; Fig. 4) (3). CymR is the master regulator of cysteine metabolism, repressing the expression of genes that lead to cysteine biosynthesis, and thus controls the response of the organism to a sulfur source. Increased exogenous sulfide levels lead to increased levels of intracellular sulfide (29) and LMW thiol persulfides (Fig. 5C). These sulfide-repressed genes include the operon NWMN_0115 to NWMN_0119, which is thought to encode a taurine (sulfonate) importer (NWMN_0116 to NWMN_0118); an operon encoding an uncharacterized ABC transporter, which is proposed to be involved in glutathione assimilation (NWMN_0143 to NWMN_0146) (3); a gene encoding a cysteine synthase (*cysK*); and genes encoding two enzymes of the transsulfuration pathway, CBS (encoded by *cysM*) and CSE (*metB*), that allow sulfur assimilation from the major human thiol, homocysteine. In addition, the expression levels of genes encoding methionine (NWMN_0246 to NWMN_0428) and cystine (NWMN_0374 and NWMN_1749 to NWMN_1751) ABC transporters and an operon (NWMN_1951 to NWMN_1952) encoding a TusA-like sulfurtransferase and a putative thiosulfate importer are also repressed (3). Loss of *cymR* in a *cymR* mutant results in overexpression of the CymR regulon, which is a transcriptomic response that is opposite the repression of the CymR regulon that we observed with exogenous sulfide. Two other genes that are strongly repressed and not part of the CymR regulon are NWMN_0557 and NWMN_0558, encoding a putative flavin adenine dinucleotide (FAD)-dependent oxidoreductase (candidate dihydrolipoamide dehydrogenases) and an Rrf2 family repressor (distinct from *cymR*; NWMN_1528 [5]) that is related to SaiR, which was recently characterized in *Bacillus subtilis* (51). SaiR regulates the expression of Spx, an activator of the response to toxic oxidants; this is consistent with the orthogonal nature of the cellular response to RSS relative to ROS (see above).

### Unregulated *cst* operon expression results in repression of staphylococcal toxin genes.

Further consideration of the genes that are downregulated in the Δ*cstR* strain (Table 4) revealed uniform repression of the expression of staphylococcal exotoxins, encoded by genes NWMN_0388 to NWMN_0397 (*ss1nm* to *ss10nm*). There is also repression of additional toxin genes, including NWMN_1075 to NWMN_1077 and the gene encoding exfoliative toxin A (*eta*), a major histocompatibility complex (MHC) class II analog that impacts T-cell function and proliferation, and a gene encoding a formyl peptide receptor-like 1 inhibitory factor which inhibits the activation of neutrophils and monocytes. In addition, genes responsible for extracellular adhesion, including those encoding clumping factor A (*clfA*) and an extracellular matrix protein (*ssp*), and genes required for anaerobic growth and antibiotic resistance are also repressed. Some of these genes are direct targets of MgrA, which harbors a single regulatory cysteine residue characterized by a range of oxidative modifications in cells (52, 53).

Other genes that are downregulated in the Δ*cstR* strain are upregulated during anaerobic growth of wild-type cells (COL) (54), while a significant fraction of these genes are similarly repressed upon treatment of *S. aureus* Newman cells with antimicrobial peptides (55). These genes include those encoding the formate acetyltransferase (*pflB*) and the formate acetyltransferase-activating enzyme; a formate-nitrate transporter (NWMN_0247) and the nitrite reductase transcriptional regulator (*nirR*); and the anaerobic ribonucleotide reductase (*nrdGD*). In addition, genes encoding a candidate flavohemoglobin (NWMN_0175), NO-inducible l-lactate dehydrogenase (*ldh1*), and l-lactate permease 2, as well as alcohol dehydrogenase (*adh1*), and genes associated with amino acid metabolism are also significantly repressed in the Δ*cstR* strain.

Comparison of the Δ*cstR* strain and the wild-type strain results seen at times shortly following an acute-phase sulfide shock shows that their transcriptomic responses diverged considerably beyond the approximately 10 genes that were similarly upregulated or downregulated under both conditions (Fig. 2A and Tables 1 to 4). Sulfide-stressed cells differ strongly from the Δ*cstR* strain in the relative concentrations of low-molecular-weight thiol persulfides (organic RSS) (26), which are strongly elevated relative to the Δ*cstR* strain but reduced relative to the untreated wild-type strain (see below). This suggests the possibility that ambient RSS might directly impact gene expression through oxidative modification of one or more cysteine-containing global regulators, with genes required for infection, dissemination, adhesion, antibiotic resistance, and anaerobic growth largely repressed in the Δ*cstR* strain relative to the wild-type strain.

**TABLE 3:** List of genes significantly (≥3.5-fold) upregulated in the Δ*cstR* strain relative to the wild-type strain

Locus tag (Newman)	Locus tag (N315)	Gene name	Fold induction	Function	Sulfide stress?^a^
NWMN_0025	SA0079		15.7	Hypothetical	−
NWMN_0026	SA0080	*tauE*	43.0	Putative sulfonate/TS effluxer	−/+^b^
NWMN_0027	SA0082	*cstA*	70.2	Multidomain sulfurtransferase^a^	+
NWMN_0028	SA0083	*cstB*	46.5	Persulfide dioxygenase-sulfur transferase^b^	+
NWMN_0029	SA0084	*sqr*	40.2	Sulfide-quinone reductase^b^	+
NWMN_0031	SA0087		5.8	Hypothetical	−
NWMN_0113	SA0162	*aldA*	5.3	Aldehyde dehydrogenase-like	+
NWMN_0151	SA0206		7.7	Putative ABC sugar transporter (NWMN_0151–0154)	−
NWMN_0329	SA0325		5.2	Glycerol-3-phosphate transporter	−
NWMN_1207	SA1140	*glpF*	16.7	Glycerol uptake facilitator protein	+
NWMN_1224			4.1	Hypothetical (82 residues)	−
NWMN_1378	SA1301	*ndk*	3.9	Nucleotide diphosphate kinase	−
NWMN_1415	SA1339	*marR*	4.2	Maltose operon repressor	+
NWMN_1658	SA1585	*putA*	5.6	Proline dehydrogenase	+
NWMN_1674	SA1601		4.1	Camphor resistance protein CrcB	−
NWMN_1681	SA1609	*pckA*	3.5	Phosphoenolpyruvate carboxykinase	−
NWMN_2074	SAS074		7.4	Hypothetical (conserved; 86 residues)	−
NWMN_2318	SA2207	*hlgA*	5.2	Gamma-hemolysin component A	−
NWMN_2402	SA2294	*gntK*	6.1	Gluconate kinase	+
NWMN_2403	SA2295	*gntR*	5.4	Gluconate operon repressor	+
NWMN_2408	SA2300		12.0	Hypothetical; putative transporter protein (glucaronic acid)	−
NWMN_2510	SA2406		6.2	Glycine betaine aldehyde dehydrogenase GbsA	−
NWMN_2513	SA2408		3.7	Putative choline transporter	−

a See Table 1.

b At least 2-fold induction with NaHS (see Table S1A; adjusted *P* value of ≤0.05).

**TABLE 4:** List of genes significantly (≥3.5-fold) downregulated in the Δ*cstR* strain relative to the wild-type strain

Locus tag (Newman)	Locus tag (N315)	Gene name	Fold induction	Function	Sulfide stress?^a^
NWMN_0022	SA0022		5.3	Hypothetical (5′-nucleotidase family)	−
NWMN_0125	SA0175		3.5	Hypothetical	−
NWMN_0162	SA0218	*pflB*	21.7	Formate acetyltransferase	−
NWMN_0163	SA0219		7.2	Hypothetical	−
NWMN_0175	SA0231		4.1	Flavohemoprotein	−
NWMN_0176	SA0232	*ldh1*	34.0	l-Lactate dehydrogenase	−
NWMN_0247	SA0293		6.0	Formate/nitrite transporter family protein	−
NWMN_0388	SA0382	*ss1nm*	9.2	Superantigen-like protein 1	−
NWMN_0389	SA0383	*ss2nm*	8.5	Superantigen-like protein 2	−
NWMN_0390	SA0384	*ssl3nm*	22.6	Superantigen-like protein 3	−
NWMN_0391		*ssl4nm*	79.3	Superantigen-like protein 4	−
NWMN_0392	SA0386	*ss5nm*	9.2	Superantigen-like protein 5	−
NWMN_0393		*ssl6nm*	11.9	Superantigen-like protein 6	−
NWMN_0394	SA0387	*ssl7nm*	12.5	Superantigen-like protein 7	−
NWMN_0395	SA0388	*ssl8nm*	5.9	Superantigen-like protein 8	−
NWMN_0396	SA0389	*ssl9nm*	10.3	Superantigen-like protein 9	−
NWMN_0397	SA0390	*ssl10nm*	6.4	Superantigen-like protein 10	−
NWMN_0426	SA0420		3.6	ABC transporter: amino acid (ATPase)	+
NWMN_0436	SA0430	*gltB*	3.9	Glutamate synthase, large subunit	−
NWMN_0577	SA0562	*adh1*	24.5	Alcohol dehydrogenase (eukaryote-like)	−
NWMN_0756	SA0742	*clfA*	4.1	Clumping factor A	−
NWMN_0758	SA0744	*ssp*	6.9	Extracellular matrix; plasma binding (cell wall)	−
NWMN_1067	SA1001		4.8	Formyl peptide receptor-like 1 inhibitory factor^b^	−
NWMN_1075	SA1009		8.1	Superantigen-like protein (toxin c)	−
NWMN_1076	SA1010		10.6	Superantigen-like protein	−
NWMN_1077	SA1011		11.8	Superantigen-like protein	−
NWMN_1082	SA1016	*eta*	3.8	Exfoliative toxin A	−
NWMN_1346	SA1269		5.5	Hypothetical (membrane efflux)	−
NWMN_1347	SA1270		7.2	Amino acid permease	−
NWMN_1348	SA1271	*ilvA*	12.9	Threonine dehydratase	−
NWMN_1349	SA1272	*ald*	22.8	Alanine dehydrogenase	−
NWMN_1749	SA1674		3.9	Glutamine transport	−
NWMN_1850	SA1728	*nadE*	3.8	NAD synthetase (glutamine or ammonia dependent)	−
NWMN_1872	SA1751	*map*	5.4	MHC class II analog protein	−
NWMN_2268	SA2156		12.7	l-Lactate permease 2	−
NWMN_2301	SA2189	*nirR*	10.3	Nitrite reductase transcriptional regulator^c^	−
NWMN_2375	SA2266		5.0	NAD short-chain dehydrogenase/reductase (SDR)	−
NWMN_2377	SA2268		4.6	Hypothetical (63 residues)	−
NWMN_2412	SA2302		3.7	ABC transporter (ATPase); lantibiotic	−
NWMN_2448	SA2336	*clpC*	5.0	Clp protease C subunit (ATPase subunit)	−
NWMN_2465	SA2352		5.2	Hypothetical (NWMN_2365–2361 operon)	−
NWMN_2473	SA2360		3.8	Hypothetical (76 residues)	−
NWMN_2514	SA2409	*nrdG*	4.5	Ribonucleotide reductase, anaerobic (small subunit)	−
NWMN_2515	SA2410	*nrdD*	5.9	Ribonucleoside-triphosphate reductase, anaerobic	−

a See Table 2.

b Secreted protein that specifically inhibits the activation of neutrophils and monocytes by binding to the formylated peptide receptor and the C5a receptor; blocks neutrophil migration toward the infection site, and hinders the establishment of the initial defense against the infection.

c *nirBG* and *narGHJ* expression downregulated by approximately 2-fold (Table S1A).

### Transcriptomic profiling of the effects of an exogenous HNO donor, Angeli’s salt.

Emerging evidence suggests that many of the properties attributed to H_2_S as a signaling molecule may derive from a significant increase in the levels of organic and inorganic polysulfide species and, in some cases, of HNO (15). HNO reacts rapidly with LMW (and protein) thiols (10, 19). This leads to disulfide bond formation in the presence of resolving thiol with the release of hydroxylamine (10) and sulfinamides [RS(O)NH_2_], which are in turn slowly reduced by cellular thiols (56). Indeed, baker’s yeast encodes an enzyme that catalyzes the NADPH-dependent reduction of the *S*-nitrosoglutathione-derived glutathione sulfinamide to reduced glutathione, which accumulates in cells under conditions of NO· stress (57). We therefore tested the effects of HNO added to aerobically growing cells by the use of Angeli’s salt (AS), i.e., dinitrogen trioxide dianion (Na_2_N_2_O_3_), which undergoes cleavage to yield HNO and nitrite (NO_2_^−^) (Fig. 1C). In contrast to the minimal induction (Fig. S5) or absence of induction of the *cstR*-regulated genes resulting from the addition of an NO· donor or nitrite, respectively (29), we found that AS significantly induced *cstA* expression in a quantitative reverse transcription-PCR (qRT-PCR) experiment (Fig. 5A). Further, a Δ*cstR* strain complemented with a mutant CstR unable to sense persulfides cannot be induced under the same conditions, suggesting that HNO impacts CstR function. These cells also exhibit a dramatic growth defect relative to the wild-type strain stressed with HNO (Fig. 5B). We next tested if HNO is capable of reacting directly with CstR thiols, leading to disulfide bond formation (10), which would induce derepression of CstR-regulated genes (29). Treatment of reduced CstR with Angeli’s salt *in vitro* does indeed yield CstR characterized by an interprotomer disulfide bond between C31 and C60′ confirmed by both liquid chromatography-electrospray ionization-mass spectrometry (LC-ESI-MS) and LC-tandem MS (LC-MS/MS), with no cross-linked products obtained upon incubation with sodium nitrite (Fig. S6). This reveals that HNO is capable of impacting CstR-regulated transcription directly by forming a cross-linked CstR which has lower affinity for operator DNA (28).

10.1128/mSphere.00082-17.5 FIG S5  Measurement of the expression levels of *cstA* in the *cst* operon in cells treated with 0.5 mM methylamine hexamethylene methylamine NONOate (MAHMA NONOate) by qRT-PCR. *cstA* expression was seen 10 min or 30 min postexposure relative to unstressed cell results. Error bars represent standard deviations of results of triplicate biological experiments, with statistical significance established using a paired *t* test relative to untreated wild-type cell results (0 min) (*, *P* < 0.05; n.s., no significant change). Download FIG S5, EPS file, 0.6 MB. Copyright © 2017 Peng et al. 2017 Peng et al. This content is distributed under the terms of the Creative Commons Attribution 4.0 International license.

10.1128/mSphere.00082-17.6 FIG S6  CstR reacts with AS to form cross-linked CstR characterized by an interprotomer disulfide bond between C31 and C60′. (A) CstR was mostly in reduced form. However, the cross-linked CstR was largely observed after the reaction with 20-fold molar excess AS (B) but not in the reaction with nitrite (C), another product of AS decomposition. (D) The formation of a disulfide bond between C31 and C60′ was confirmed by LC-MS/MS. The expected *m/z* of reduced CstR monomer is 9,641.2. Electron transfer dissociation (ETD) mode was used for the sequencing analysis. Download FIG S6, EPS file, 2.9 MB. Copyright © 2017 Peng et al. 2017 Peng et al. This content is distributed under the terms of the Creative Commons Attribution 4.0 International license.

**FIG 6:**
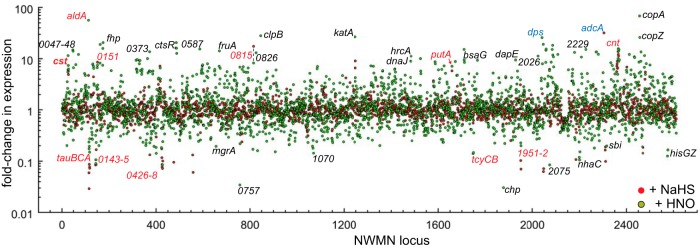
RNAseq transcriptomic analysis of *Staphylococcus aureus* strain Newman cells treated with sulfide (red filled circles) versus Angeli’s salt (green filled circles). The fold change in expression for each locus tag is indicated (Tables 1 to 2; Table S1A). Gene names are indicated where known. Expression of those highlighted in red text was significantly induced or repressed under both experimental conditions; expression of those highlighted in light blue was induced during calprotectin (CP) and Angeli’s salt (nitroxyl) treatment.

**FIG 7:**
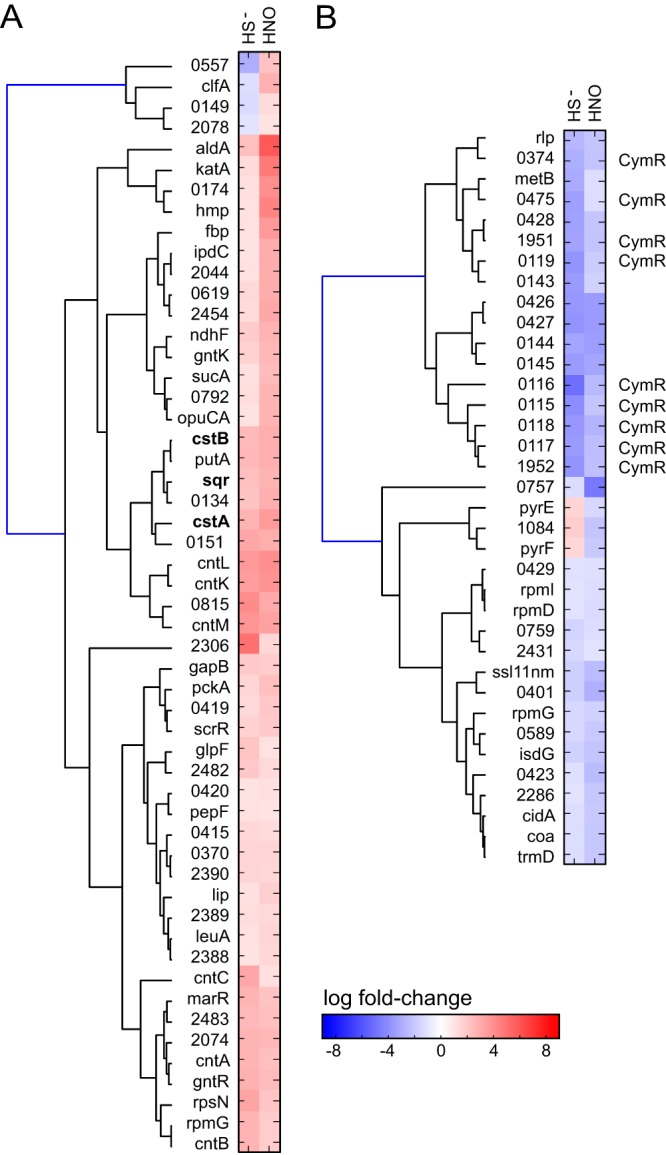
Clustering analysis of the RNAseq results based on pairwise comparisons of gene expression in sulfide (HS^−^)-treated versus AS (HNO)-treated *S. aureus* strain Newman cells. (A) Genes that were upregulated by HNO compared to the analogous change in HS^−^-treated cells. (B) Genes that were downregulated by HNO compared to the analogous change in HS^−^-treated cells. Genes of the *cst* operon are in boldface (panel A), while those genes previously identified as part of the CymR regulon (3) are marked "CymR" (panel B). Genes are clustered according to similarity in change in expression in each pair of experiments. Only the genes that were affected by both sulfide and AS stress are shown here (Tables 1 and 2, Fig. S9, and Table S1A for a compilation of all transcriptomic changes observed under these conditions).

A transcriptomic analysis of all genes induced (Table 1) or repressed (Table 2; see Table S1A for a complete list) by AS reveals a significant genome-wide change in cellular transcription (Fig. 6). We focused our attention on those genes whose expression changed by approximately 1 standard deviation from the mean induction level (approximately 10-fold; Table 1) or the mean repression level (approximately 5-fold; Table 2). Beyond the *cst* operon, there was a subset of genes that were upregulated by both HNO and sulfide treatment relative to the results seen with untreated wild-type cells (Table 1 and Fig. 7). These genes included a gene that is part of the CymR regulon, *aldA* (NMWN_0113), which is among the most highly (56-fold) upregulated genes in the genome; *cntKLM*, encoding a broad-spectrum metallophore biosynthetic cluster (48) and among the genes most highly upregulated by sulfide treatment (Table 1); and NWMN_0815, a gene encoding a putative pyridine nucleotide (FAD) disulfide reductase (Fig. 7). These genes are also associated with the zinc limitation, CP-mediated transcriptomic response (Table 1) (Fig. 2), which suggests that zinc may well become limiting as a result of intracellular chelation of the metal upon AS treatment (58). Several other genes involved in the metal limitation response, e.g., *rpmG2*, *rpsN2*, *cntAB*, and NWMN_2483, are also detectably induced by HNO stress but to a level that is lower than that due to sulfide stress. Consistent with this, AS treatment leads to a detectable (approximately 3-fold) decrease in the Zn concentration and to a smaller (1.8-fold) (Fig. S4B) change in the Ni concentration in the growth medium, suggesting that HNO, like sulfide itself, may be capable of reducing the bioavailability of transition metals in cells.

The remaining genes in the far larger panel that are upregulated by AS treatment but unaltered by sulfide treatment show some overlap with respect to those genes induced by nitrite in nitrate-respiring, nitrite-stressed cells (59). Most notable is the massive upregulation of the *copAZ* operon, encoding a Cu(I)-transporting P-type ATPase effluxer and a Cu(I) chaperone, both under the transcriptional control of Cu(I)-sensing repressor CsoR (NWMN_1992) (28). CsoR is a paralog of CstR and is unresponsive to sulfide stress in cells (28). This suggests that HNO-mediated modification of LMW thiols, which maintain bioavailable Cu(I) at low levels, may result in the displacement of Cu(I) from these stores, which is then sensed by CsoR. Indeed, analysis of total cell-associated metal levels by inductively coupled plasma-mass spectrometry (ICP-MS) reveals a significant and specific increase in levels of cellular Cu (Fig. S7), consistent with this hypothesis. The remaining AS-affected genes represent an Fe overload response, the origin of which is probed below, coupled with a PerR-regulated ROS response and induction of the stress-associated CtsR regulon (*clpB*, *clpC*) and a DNA damage response (*uvrBC*) (Fig. S2). These transcriptomic changes, taken collectively, are consistent with an acute-phase combined ROS/RNS stress response induced by AS treatment (Fig. S2).

10.1128/mSphere.00082-17.7 FIG S7  Analysis of cellular metal before and after treatment with 0.2 mM AS (A) and 0.2 mM sodium nitrite (B). ICP-MS was used to quantify the concentrations of transition metal ions in the untreated cell (gray bars) or in the cell treated with 0.2 mM AS or nitrite (light blue bars). (A) AS treatment did not lead to a significant change in the total levels of cellular transition metals, with the exception of Cu, whose level increased approximately 4-fold. (B) Nitrite treatment with the same concentration led to no significant change in all cellular metals. Download FIG S7, EPS file, 0.9 MB. Copyright © 2017 Peng et al. 2017 Peng et al. This content is distributed under the terms of the Creative Commons Attribution 4.0 International license.

AS also represses a number of virulence factors, including superantigen-like proteins; secreted von Willebrand factor-binding protein; proteins encoded by NWMN_1066 and NWMN_1068 to NWMN_1070, which are predicted to be fibrinogen binding, chemotaxis-inhibiting proteins; secretory antigen precursor SsaA; and immunoglobulin G-binding protein. We globally compared those genes whose expression is affected by AS to genes encoding other virulence factor regulons, including those encoded by *sarA* (60), *sarZ* (61), *mgrA* (62), *sigB* (63), and *rot* (64). We generally found no clear correlation of upregulation or downregulation between AS-responsive genes and those encoding these virulence regulons, with the exception of *sigB*, where many of the transcriptomic changes that were common under these two conditions occurred in the same direction (Fig. S8). SigB is an alternative sigma factor (σ^B^) that controls the response to heat stress, oxidative stress, and antibiotic stress and appears to be linked to intracellular survival during chronic infection (65) while impacting the expression of genes encoding other virulence regulators, including *sarA*, *sarS*, and *arlRS*, a two-component system of autolysis (63). We note that most of the virulence factors repressed by AS treatment are not regulated by sulfide stress, suggesting that host NO·-derived RNS, e.g., HNO (Fig. 1B), likely have important regulatory roles in virulence expression distinct from that of sulfide.

10.1128/mSphere.00082-17.8 FIG S8  Analysis of all transcriptomic (RNAseq) changes in the sulfide-stressed (HS^−^) and AS-treated cells compared to the regulons of selected virulence factors. Genes associated with the *sarA* (A) (60), *sarZ* (B) (61), *mgrA* (C) (62), *sigB* (D) (63), and *rot* (E) (64) regulons are shown. The cutoff is 2-fold change. Gene names are shown (where known) on the right. Note that only genes that are significantly regulated by either sulfide or AS stress or both sulfide and AS stress and the indicated virulence factor regulon are shown in each panel. With the exception of the *sarZ* and *mgrA* experiments, the transcriptomic profiling experiments were carried out using post-exponential-phase or stationary-phase cultures, which are different from the growth conditions used in our RNAseq experiments. (A) *sarA*: the 48 genes shown represent the 40% of the transcriptome that is affected by *sarA*, corresponding to 12% of upregulated and 2% of downregulated genes that change in the same direction. (B) *sarZ*: the 33 genes shown represent the 57% of the transcriptome that is affected by *sarZ*, corresponding to 0% of upregulated and 21% of downregulated genes that change in the same direction. (C) *mgrA*: the 53 genes shown represent the 15% of the transcriptome that is affected by *mgrA*, corresponding to 5% of upregulated and 2% of downregulated genes that change in the same direction. (D) *sigB*: the 136 genes shown represent the 54% of the transcriptome that is affected by *sigB*, corresponding to 56% of upregulated and 32% of downregulated genes that change in the same direction. (E) The 47 genes shown represent the 32% of the transcriptome that is affected by *rot*, corresponding to 19% of upregulated and 12% of downregulated genes that change in the same direction. Download FIG S8, PDF file, 0.6 MB. Copyright © 2017 Peng et al. 2017 Peng et al. This content is distributed under the terms of the Creative Commons Attribution 4.0 International license.

### RSS profiling in sulfide-stressed versus Angeli’s salt-stressed cells.

We previously employed a fluorescence-based analytical method to detect and quantify LMW monobromobimane (mBBr)-derivatized sulfur-containing metabolites in cells (29). Here, we extended this method to incorporate ratiometric (^32^S/^34^S) tandem mass spectrometry, in which the concentrations of all organic thiols and of per- and polysulfides relative to those seen with an internal standard can be detected in a single experiment. We quantified bacillithiol persulfide (BSSH), cysteine persulfide (CysSSH), and coenzyme A persulfide (CoASSH) in the wild-type and *ΔcstR S. aureus* strains and in sulfide- or AS-stressed wild-type *S. aureus*. The *ΔcstR* strain showed slightly lower levels of LMW persulfides (Fig. 5C), consistent with an RSS clearance function of the *cst* operon-encoded enzymes. As predicted from the transcriptomics experiments, both sulfide stress (Fig. 5C) and AS treatment (Fig. 5D) caused an increase in cellular levels of LMW thiol persulfides, while nitrite, one of the AS decomposition products (Fig. 1B and C), did not (Fig. 5E). These results directly implicate HNO or a downstream reaction product(s) in this cellular increase in RSS (Fig. 1B). Thus, HNO may directly react with CstR thiols to induce transcriptional derepression of the *cst* operon (Fig. S6) or, alternatively, may induce higher levels of cellular LMW persulfides which are in turn sensed by CstR.

To further explore this, we reasoned that HNO could directly mediate an increase in cellular concentrations of RSS via at least two possible mechanisms. One is a mechanism by which HNO upregulates the expression of a sulfide biogenesis pathway(s), e.g., one involving CBS (CysM) and CSE (MetB). An alternative possibility is that AS-derived HNO disassembles Fe-S clusters directly or reacts with molecular oxygen to create ONOO^–^ (21, 66), which is known to be capable of destabilizing Fe-S clusters in proteins (Fig. 1C) (24). Both would lead to an increase in endogenous sulfide as well as chelatable Fe(II) levels in the cell. Human CBS binds heme via coordination by Cys52 and His65 in an N-terminal heme-binding domain, and RNS are capable of regulating CBS activity by changing the oxidation state of the heme (67). However, this domain is not conserved in *S. aureus* CysM (alignment not shown). Consistent with this, RSS levels are in fact transiently elevated in a Δ*cysM* Δ*metB* strain by AS treatment, albeit to an extent lower than that seen in the wild-type strain (Fig. 5F).

To measure the effect of molecular oxygen on this AS (HNO) stress-induced increase in RSS, we carried out this experiment under microaerophilic conditions, where oxygen levels are significantly decreased by growing static cultures in tightly capped tubes, with 5 mM nitrate as the electron acceptor of anaerobic respiration. AS-inducible increases in RSS were observed in these cultures (Fig. 5G) that were nearly equal to those seen with aerobically grown cells (Fig. 5D). Cells grown aerobically and treated with a burst of ONOO^–^ also induced organic RSS but to a level that was lower overall than that seen with AS treatment (Fig. 5H). This result may have been related to the vanishingly short lifetime of ONOO^–^ under these conditions. We propose that the presence of HNO or a downstream product(s) (Fig. 1B) leads to the disassembly of Fe-S clusters and to a corresponding increase in endogenous sulfide and RSS levels, with the oxidant ONOO^–^ also capable of this chemistry (21, 66). In support of this idea, although the total cell-associated Fe level did not change (Fig. S7), our transcriptomic analysis strongly suggests an increase in the level of cytoplasmic chelatable iron, as exemplified by the upregulation of genes encoding the Fe-S cluster biogenesis pathway (*sufC*, *sufD*, *sufS*, *NIFU*, and *sufB*), the iron-storage proteins ferritin (*ftn*) and Dps (*dps*), and the iron uptake repressor (*fur*; NWMN_1406), which could chelate cellular Fe and further repress Fe import. Regardless of the precise mechanism involved, these data, taken collectively, suggest that HNO, as a principal product of H_2_S/NO· interplay (15, 68), directly impacts endogenous sulfur speciation and global gene expression in *S. aureus*.

### Conclusions.

In this report, we show that the cellular transcriptomic response of the major human pathogen *S. aureus* to the effects of exogenous sulfide exhibits some parallels to the cellular response to Angeli’s salt, a commonly used HNO donor. Under these conditions, HNO appears to signal partly through perturbations in sulfur speciation in cells, as anticipated by much of the small-molecule chemistry that has been reported for this primary product of RNS/RSS interplay (15, 68). The origin of both effects derives in part from a significant increase in levels of cellular RSS (Fig. 5). The RSS-regulated transcriptomic response is opposite that induced by oxidants and suggests the possibility that bacterial cells can manage intracellular RSS as a means to provide protection against irreversible oxidation by oxidative stressors, as has been previously established in mammalian cells (25, 69). In contrast, decreases in levels of ambient RSS induced by overexpression of the *cst*-encoded sulfide oxidation system in a Δ*cstR* strain repressed virulence gene expression, i.e., expression of genes required for infection, dissemination, and adhesion to cells.

These results support the use of cellular RSS as a readily deployable chemical strategy to impact the physiological state of a bacterial community (32) and otherwise to mediate an adaptive response to changes in host microenvironments mediated by ROS and RNS. Indeed, multiple reactive small-molecule stressors often have a synergistic effect on microbial killing (70) and it is unusual for a pathogen to encounter a single stressor at a site of infection. This deployment of RSS, however, must be tightly managed, as intracellular sulfide and HNO (or downstream reaction products) significantly reduce zinc bioavailability, while HNO potentially increases levels of cytoplasmic free Cu (Fig. S7) and free Fe, the latter possibly via destruction or inhibition of assembly of Fe-S clusters. It is interesting in this context that one of the *cst* operon-encoded proteins, CstA, is capable of stripping the active site persulfide from the major *S. aureus* cysteine desulfurase SufS in a persulfide transfer reaction (30). This might provide a means to divert sulfur flow from Fe-S protein biogenesis to cellular RSS as a protective mechanism to minimize exposure to oxidative damage mediated by free Fe. This is consistent with the strong upregulation of Fe- and PerR-regulated ROS-inducible genes, including those encoding catalase (*katA*), flavohemoglobin (*hmp*), a candidate peroxiredoxin (*bcp*), alkylhydroperoxidases (*ahpD*, *ahpF*), a candidate nitroreductase, and the iron-storage protein Dps (*dps*) (Table S1A; Table 1; Fig. 7). These studies have set the stage for further elucidation of H_2_S/NO cross talk and proteome *S*-sulfhydration-based pathways in aerobically versus anaerobically growing bacterial cells.

10.1128/mSphere.00082-17.9 FIG S9  Clustering analysis that summarizes all transcriptomic (RNAseq) changes in the Δ*cstR*, sulfide-stressed (HS^–^), AS (HNO)-treated, and calprotectin (CP)-treated cells that showed significant pairwise changes in expression. This figure recapitulates the data presented in Fig. 2 and 7 in the main text but provides more detail. A listing of all transcriptomic changes is provided in Table S1A. Locus tags are indicated on the left, with gene names shown (where known) on the right. Additional description is provided on the far right; the absence of an entry indicates a hypothetical protein of unknown function. Download FIG S9, PDF file, 0.6 MB. Copyright © 2017 Peng et al. 2017 Peng et al. This content is distributed under the terms of the Creative Commons Attribution 4.0 International license.

## MATERIALS AND METHODS

### Chemicals and reagents.

AS (82230) and sodium peroxynitrite (81565) were purchased from Cayman Chemical. Monobromobimane (M-20381) was purchased from Invitrogen. Sulfur-34 metal (SLM-1085-PK) used for Na_2_^34^S synthesis was purchased from Cambridge Isotope Laboratories, Inc. Sodium sulfide (407410), sodium nitrite (237213), sodium nitrate (S5506), oxidized coenzyme A (C2643) used to synthesize the CoASSH standard, l-cystine (C8755) used to synthesize the CysSSH standard, and other reagents were purchased from Sigma-Aldrich.

### *S. aureus* RNAseq and qRT-PCR experiments.

Sample collection, RNA extraction, and the procedure for qRT-PCR experiments were described previously (29). The primers used in qRT-PCR experiments are listed in Table S1B in the supplemental material. Sequencing reads were trimmed using Trimmomatic (version 0.33 [71]) with the following parameters: ILLUMINACLIP:adapter.fa:2:20:6 LEADING:3 TRAILING:3 SLIDINGWINDOW:4:20 MINLEN:35. The trimmed reads were mapped onto the *Staphylococcus aureus* subsp. *aureus* strain Newman genome using bowtie2 (version 2.1 with default parameters) (72). Read counts for genes and intergenic intervals were calculated using a custom perl script. The resulting gene/interval counts were used to conduct differential expression analysis using the program DESeq2 algorithm (73) with default parameters. For transcriptome sequencing (RNAseq) analysis of calprotectin (CP)-treated cultures, bacteria were cultured overnight in Chelex-treated RPMI medium plus 1% Casamino Acids supplemented with 1 mM MgCl_2_, 100 μM CaCl_2_, and 1 μM FeSO_4_. These samples were then back-diluted 1 to 100 into growth medium containing 38% tryptic soy broth (TSB)–72% calprotectin buffer (20 mM Tris [pH 7.5], 100 mM NaCl, 3 mM CaCl_2_, 10 mM β-mercaptoethanol), supplemented with 1 µM Zn and 1 µM Mn. The bacteria were grown to an exponential-phase optical density at 600 nm (OD_600_) of 0.25 to 0.4 in the presence and absence of 960 µg/ml CP. Samples were harvested and processed as previously described (29), with the exception that RNA was isolated using the method reported by Collins et al. (74). The mean level of induction of all genes that showed an increase in expression of ≥2.0-fold at an adjusted *P* value of ≤0.05 in the CP-treated cells was 20-fold (±59-fold), with a median genome-wide level of induction of 4.4-fold (330 genes). The mean level of expression for all genes that were downregulated as a result of CP treatment was 7.4-fold (±8.0-fold), with a median level of genome-wide repression of 4.3-fold (289 genes). The results of all RNAseq experiments have been deposited in the GEO database (see below).

### CstR reaction with AS.

The purification of CstR was carried out as described previously (29). A 200-µl volume of 25 µM apo- and reduced CstR was incubated anaerobically with a 20-fold molar excess over levels of thiols of sodium nitrite or Angeli’s salt in 25 mM Tris (pH 8.0)–500 mM NaCl–5 mM EDTA at room temperature. After 30 min, 100 μl of the reaction mixture was transferred to a vial, the vial was tightly capped, and the contents of the vial were injected in LC-ESI-MS experiments to determine if cross-linked CstR was formed, as previously described (29). The remainder of each sample was alkylated with iodoacetamide, digested by trypsin, and sequenced to further confirm the formation of a disulfide bond.

### Bacterial growth curves and cell culture.

The wild-type *S. aureus* Newman strain and the Δ*cstR* CstR^C31A/C60A^ strain were described previously (29). The Δ*cysM* Δ*metB* strain was constructed by allelic exchange (75). The fragment upstream of *cysM* was amplified using primers 5′-TTGAGCCTCGGAACCGGTACCAACATTAGATGGCGCCTTAG-3′ and 5′-TCCTAGCTTAGCTAGCAATTAAATCATAAGTAATCATAGATGC-3′; a spectinomycin resistance cassette was amplified from pSPC using primers 5′-GCTAGCTAAGCTAGGATCGAATCCC-3′ and 5′-GCTAGCCTAATTGAGAGAAGTTTCTATAGAATTTTTC-3′; and the fragment downstream of *metB* was amplified using primers 5′-TCTCAATTAGGCTAGCCAAGCACTAGATACTTTATAAATAATAGC-3′ and 5′-ACAGCTATGACATAGTCACGAATTCAAACACCTCTTTAACAGTTC-3′. These fragments were assembled with pKOR1 linearized by digestion with EcoRI and KpnI using NEB Gibson assembly and were integrated into the *S. aureus* genome. The genetic lesion was then transduced into a clean *S. aureus* Newman background using bacteriophage φ85, and transductants were selected with spectinomycin at 1,000 mg/liter. All bacterial strains were grown overnight in TSB with 10 μg/ml chloramphenicol. Cells were pelleted and resuspended in Hussain-Hastings-White modified (HHWm) minimal media (76) supplemented with 50 μg/ml chloramphenicol and 0.5 mM thiosulfate as the sole sulfur source. For growth curve analyses, cultures were initiated at an OD_600_ of 0.007 with or without 0.2 mM AS stress added to the growth medium. All aerobically grown cultures were grown at 37°C with shaking (200 rpm), with the OD_600_ measured every hour from h 2 to h 10.

### Quantitation of cellular LMW thiol persulfides.

Overnight *S. aureus* cells grown in TSB were diluted to an OD_600_ of 0.02 in HHWm minimal medium (76) supplemented with 0.5 mM thiosulfate as the sole sulfur source and grown aerobically. When these cultures reached an OD_600_ of 0.2, 0.2 mM disodium sulfide, AS, sodium nitrite, or sodium peroxynitrite was added. For microaerophilic conditions, 5 mM sodium nitrate was added as an electron acceptor to 50-ml tubes that were capped tightly without shaking. The tubes were opened for addition of AS and were gently inverted for mixing. Samples (5 ml plus 1 ml for protein quantification) were collected before (*t* = 0 min) and after addition of the stressor at the indicated times following addition of stressors and were centrifuged at 3,000 rpm for 10 min. The resulting pellets were washed with ice-cold phosphate-buffered saline (PBS), pelleted again by centrifugation (16,100 rpm for 5 min), and stored frozen at −80°C until use. Thawed cell pellets were resuspended in 100 μl monobromobimane (mBBr) labeling solution containing 20 mM Tris-HBr (pH 8.0), 50% acetonitrile, and 1 mM mBBr and subjected to three freeze-thaw cycles in liquid nitrogen in the dark in screw-cap tubes (77). Cell debris was removed by centrifugation, and the supernatant was transferred to a tube containing 100 μl 15 mM methanesulfonic acid (MA) to quench the labeling reaction (31). Finally, particulates were removed via passage through a 0.2-μm-pore-size centrifugal filter unit prior to injection into a liquid chromatograph mass spectrometry (LC-MS) system for quantitation of LMW thiol persulfides as follows.

Samples (10 μl) were injected into a Triart C_18_ column (YMC, Inc.) (50 by 2.0 mm inner diameter) and subjected to chromatography on a Waters Acquity Ultra Performance Liquid Chromatography (UPLC) I-class system, using a methanol-based gradient system (for solvent A, 10% methanol and 0.25% acetic acid, pH 3.0; for solvent B, 90% methanol and 0.25% acetic acid, pH 3.0) with the elution protocol at 25°C and a flow rate of 0.2 ml/min as follows: at 0 to 3 min, 0% B isocratic; at 3 to 7 min, 0% to 25% B, linear gradient; at 7 to 9 min, 25% B isocratic; at 9 to 12 min, 25% to 75% B, linear gradient; at 12 to 14 min, 75% to 100% B, linear gradient; at 14 to 14.5 min, 100% B isocratic, followed by reequilibration to 0% B. Quantitation of LMW thiols and persulfides was carried out with a Waters Synapt G2S mass spectrometer by spiking in a specific amount of authentic ^34^S-containing LMW persulfide standards synthesized with Na_2_^34^S in place of Na_2_^32^S (31) to achieve a typical ^34^S/^32^S persulfide ratio of approximately 0.2 to 1.0. To quantify the relative change in the BSH/BSSB ratio, the peak area of mBBr-derivatized BSH and the peak area of underivatized BSSB in each sample were obtained and normalized to that of a known concentration (peak area) of mBBr-labeled *N*-acetyl-cysteine (NAC), with the same concentration added to each sample. The change of the ratio was obtained by dividing the ratio seen under stress conditions by the ratio for the unstressed sample, with standard propagation of errors. Analysis of peak areas was performed in Masslynx (v 4.1) software, and the data were normalized to protein concentrations measured using a Bradford assay with bovine serum albumin (BSA) as the standard, as previously described (26). Data shown represent means and standard deviations of results from three biological replicates.

### Synthesis of isotope-labeled internal standards.

Na_2_^34^S was synthesized using a published protocol (78). Bacillithiol persulfide (BS^34^SH), cysteine persulfide (CysS^34^SH), and coenzyme A persulfide (CoAS^34^SH) were synthesized by reacting the appropriate oxidized disulfide (RSSR) with Na_2_S to obtain an equimolar mixture of the thiol RS^−^ and the persulfide RSS^–^ (16). Bacillithiol disulfide (BSSB; 5 mM), generously provided by M. Kiethly (Vanderbilt University), CoA disulfide (5 mM), or l-cystine (2.5 mM) was reacted anaerobically with a 5-fold molar excess of Na_2_^34^S–300 mM degassed phosphate buffer (pH 7.4) at 30°C for 30 min. The concentration of persulfide in the final mixture was quantified by a cold cyanolysis assay (30), and the persulfide was diluted to 0.1 mM in 20 mM Tris-HBr (pH 8.0)–50% acetonitrile–2 mM mBBr and labeled as described above. Standard samples were used without further purification.

### Transition metal measurements.

Aliquots of growth medium with or without the addition of 0.2 mM sulfide or 0.2 mM AS were taken, centrifuged to remove any precipitates, diluted 10-fold in 2.5% HNO_3_, and analyzed using a PerkinElmer Elan II DRC ICP-MS system essentially as described in our previous work (79). To analyze the total cell-associated metal content, 5-ml cultures (OD_600_ of 0.2) were pelleted, the pellet was resuspended in 400 μl 30% nitric acid, and metal concentrations were determined as described above for the growth medium, with normalization to the amount of protein in each sample (in nanomoles per milligram of protein) as previously described (80). Metal concentrations were determined from a standard curve of 1 to 30 ppb metal stock solutions.

### Accession number(s).

The results of all RNAseq experiments have been deposited in the GEO database under GenBank accession number GSE99432.
