# Supplementary figures and images for: Sulfide Homeostasis and Nitroxyl Intersect via Formation of Reactive Sulfur Species in Staphylococcus aureus
Source: mSphere. 2017 Jun 21;2(3):e00082-17. doi: 10.1128/mSphere.00082-17 (PMC5480029; doi:10.1128/mSphere.00082-17)

A

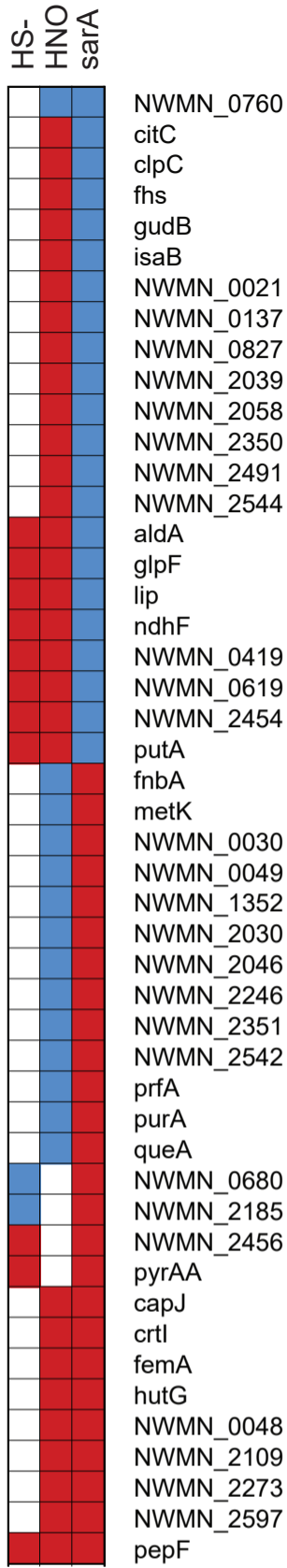

B

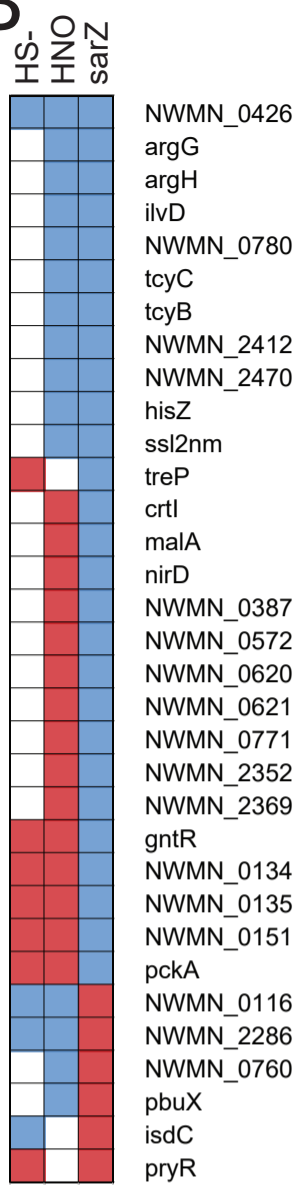

C

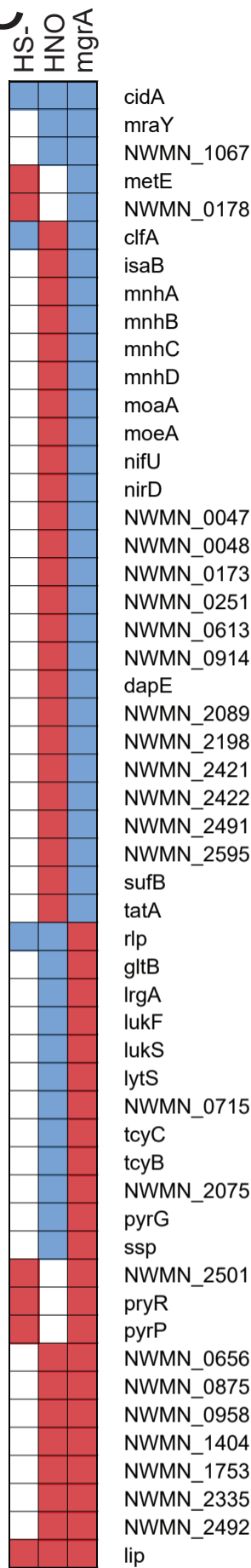

D

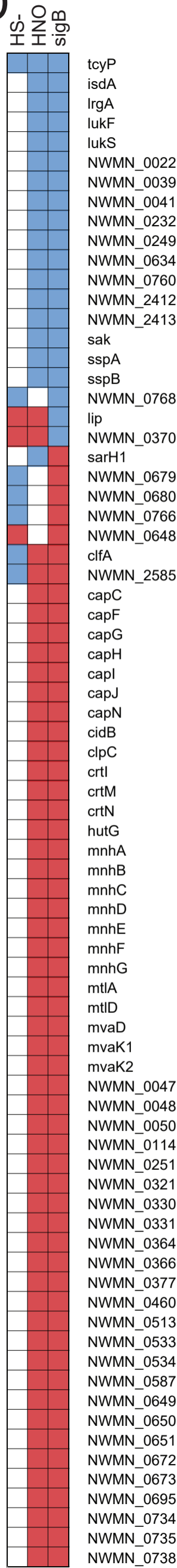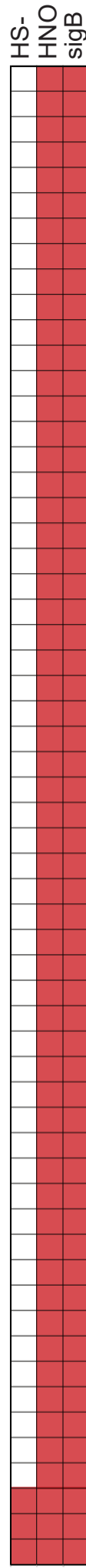

E

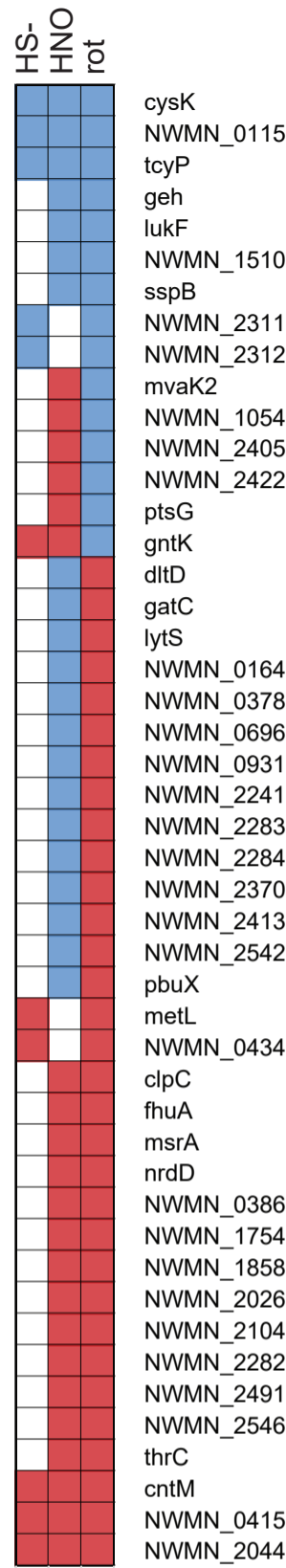☐ Change-fold<2☒ Up-regulation☒ Down-regulation

Supplement: FIG S8 [file sph003172308sf9.pdf]

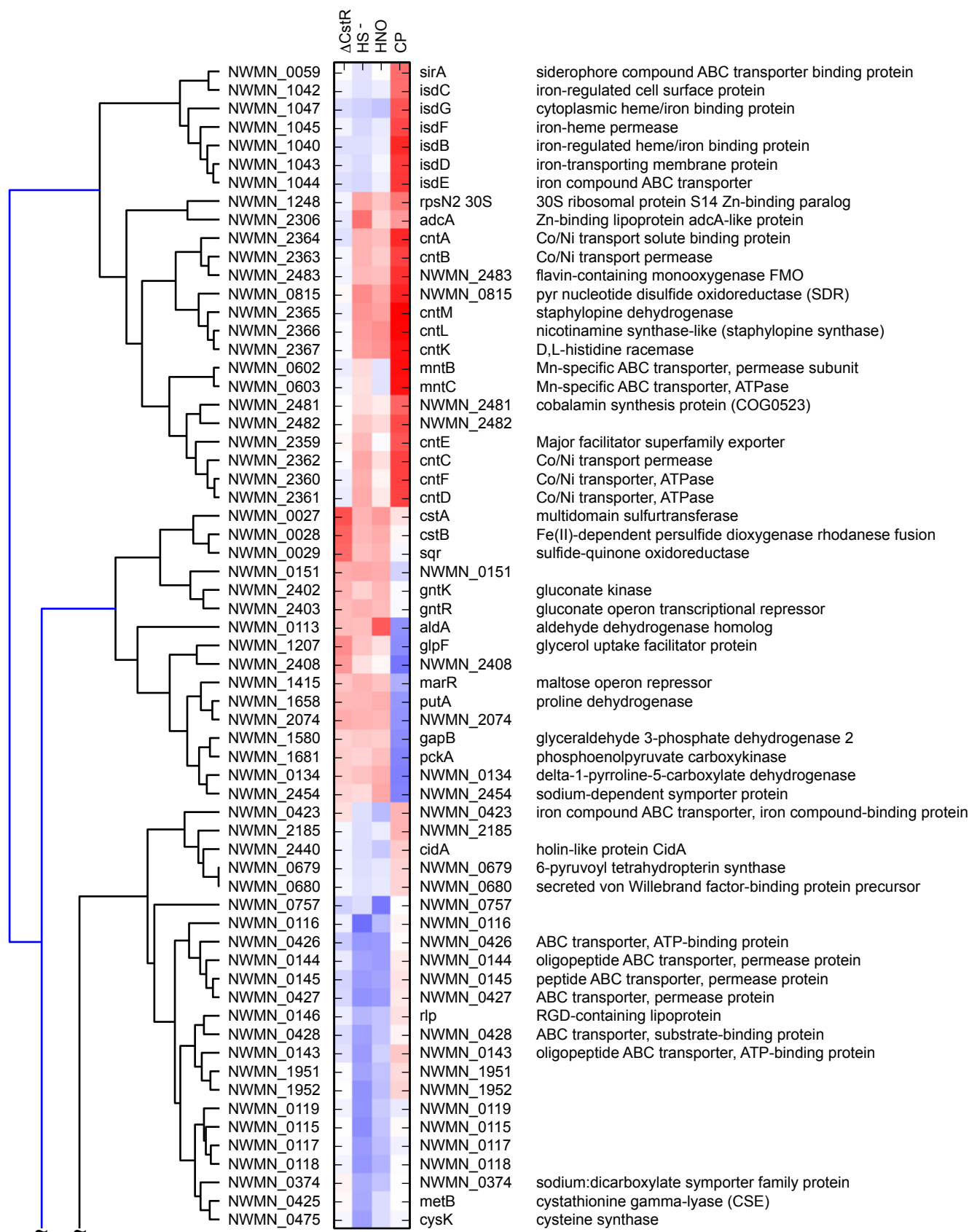

log fold-change

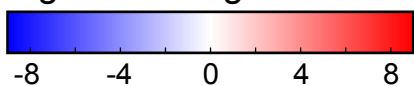

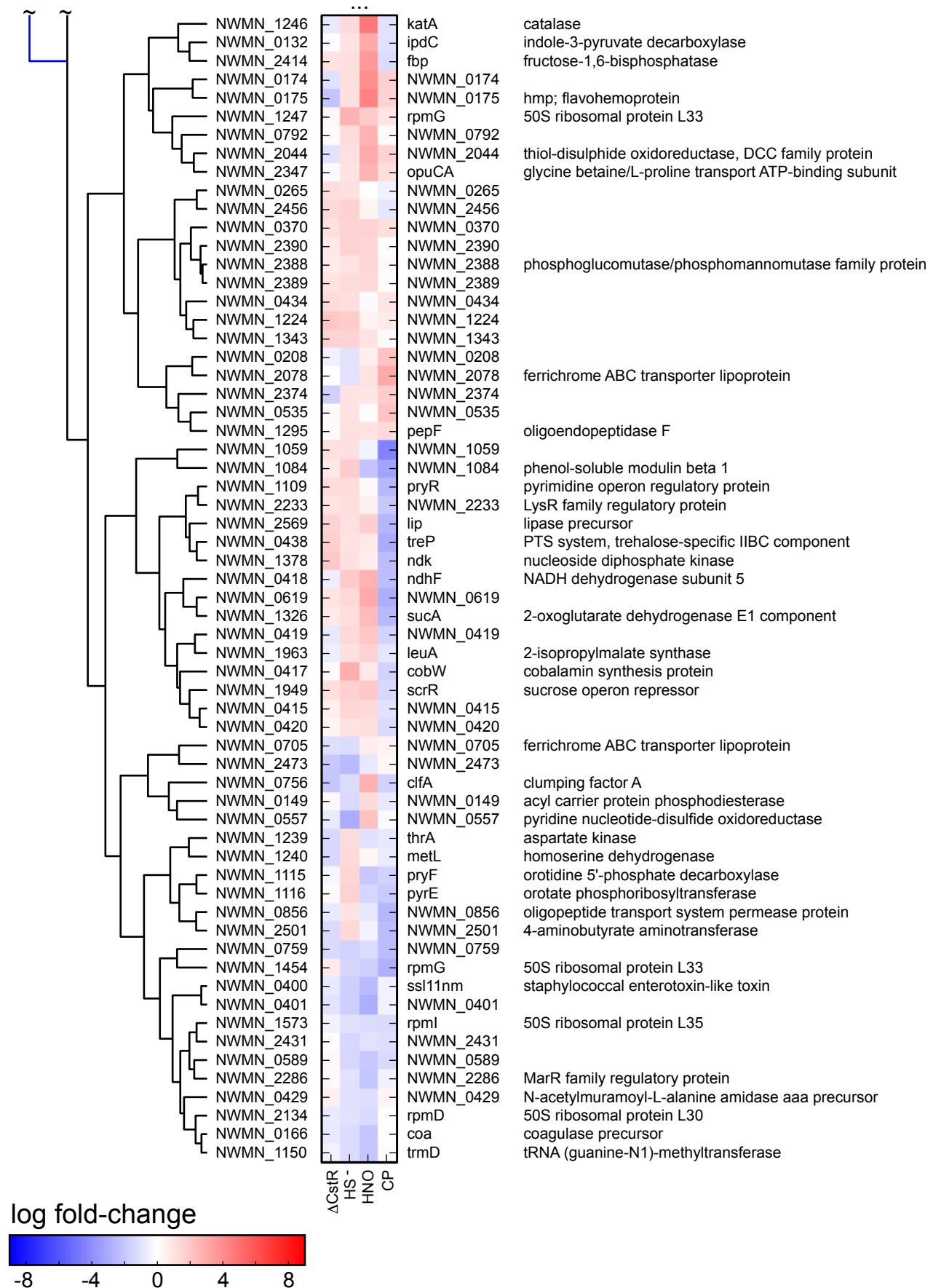

Supplement: FIG S9 [file sph003172308sf10.pdf]
